# Adeno-associated virus 2 infection in children with non-A–E hepatitis

**DOI:** 10.1038/s41586-023-05948-2

**Published:** 2023-03-30

**Authors:** Antonia Ho, Richard Orton, Rachel Tayler, Patawee Asamaphan, Vanessa Herder, Chris Davis, Lily Tong, Katherine Smollett, Maria Manali, Jay Allan, Konrad Rawlik, Sarah E. McDonald, Elen Vink, Louisa Pollock, Louise Gannon, Clair Evans, Jim McMenamin, Kirsty Roy, Kimberly Marsh, Titus Divala, Matthew T. G. Holden, Michael Lockhart, David Yirrell, Sandra Currie, Maureen O’Leary, David Henderson, Samantha J. Shepherd, Celia Jackson, Rory Gunson, Alasdair MacLean, Neil McInnes, Amanda Bradley-Stewart, Richard Battle, Jill A. Hollenbach, Paul Henderson, Miranda Odam, Primrose Chikowore, Wilna Oosthuyzen, Meera Chand, Melissa Shea Hamilton, Diego Estrada-Rivadeneyra, Michael Levin, Nikos Avramidis, Erola Pairo-Castineira, Veronique Vitart, Craig Wilkie, DIAMONDS Consortium, ISARIC4C Investigators, J. Kenneth Baillie, Malcolm G. Semple, Gail Carson, Peter J. M. Openshaw, Jake Dunning, Laura Merson, Clark D. Russell, David Dorward, Maria Zambon, Meera Chand, Richard S. Tedder, Saye Khoo, Lance C. W. Turtle, Tom Solomon, Samreen Ijaz, Tom Fletcher, Massimo Palmarini, Antonia Ho, Emma C. Thomson, Nicholas Price, Judith Breuer, Thushan de Silva, Chloe Donohue, Hayley Hardwick, Wilna Oosthuyzen, Miranda Odam, Primrose Chikowore, Lauren Obosi, Sara Clohisey, Andrew Law, Lucy Norris, Sarah Tait, Murray Wham, Richard Clark, Audrey Coutts, Lorna Donnelly, Angie Fawkes, Tammy Gilchrist, Katarzyna Hafezi, Louise MacGillivray, Alan Maclean, Sarah McCafferty, Kirstie Morrice, Lee Murphy, Nicola Wrobel, Sarah E. McDonald, Victoria Shaw, Katie A. Ahmed, Jane A. Armstrong, Lauren Lett, Paul Henderson, Louisa Pollock, Shyla Kishore, Helen Brotherton, Lawrence Armstrong, Andrew Mitra, Anna Dall, Kristyna Bohmova, Sheena Logan, Louise Gannon, Ken Agwuh, Srikanth Chukkambotla, Ingrid DuRand, Duncan Fullerton, Sanjeev Gar, Clive Graham, Tassos Grammatikopoulos, Stuart Hartshorn, Luke Hodgson, Paul Jennings, George Koshy, Tamas Leiner, James Limb, Jeff Little, Sheena Logan, Elijah Matovu, Fiona McGill, Craig Morris, John Morrice, David Price, Henrik Reschreiter, Tim Reynolds, Paul Whittaker, Thomas Jordan, Rachel Tayler, Clare Irving, Katherine Jack, Maxine Ramsay, Margaret Millar, Barry Milligan, Naomi Hickey, Maggie Connon, Catriona Ward, Laura Beveridge, Susan MacFarlane, Karen Leitch, Claire Bell, Lauren Finlayson, Joy Dawson, Janie Candlish, Laura McGenily, Tara Roome, Cynthia Diaba, Jasmine Player, Natassia Powell, Ruth Howman, Sara Burling, Sharon Floyd, Sarah Farmer, Susie Ferguson, Susan Hope, Lucy Rubick, Rachel Swingler, Emma Collins, Collette Spencer, Amaryl Jones, Barbara Wilson, Diane Armstrong, Mark Birt, Holly Dickinson, Rosemary Harper, Darran Martin, Amy Roff, Sarah Mills, Massimo Palmarini, Surajit Ray, David L. Robertson, Ana da Silva Filipe, Brian J. Willett, Judith Breuer, Malcolm G. Semple, David Turner, J. Kenneth Baillie, Emma C. Thomson

**Affiliations:** 1 Medical Research Council–University of Glasgow Centre for Virus Research, Glasgow, UK; 2 Department of Paediatrics, Royal Hospital for Children, Glasgow, UK; 3 Pandemic Science Hub, Centre for Inflammation Research and Roslin Institute, University of Edinburgh, Edinburgh, UK; 4 Department of Paediatrics, NHS Tayside, Dundee, UK; 5 Department of Pathology, Queen Elizabeth University Hospital, Glasgow, UK; 6 Public Health Scotland, Glasgow, UK; 7 West of Scotland Specialist Virology Centre, Glasgow, UK; 8 Virology Laboratory, Ninewells Hospital, Dundee, UK; 9 Histocompatibility and Immunogenetics (H&I) Laboratory, Scottish National Blood Transfusion Service, Edinburgh Royal Infirmary, Edinburgh, UK; 10 Department of Neurology and Department of Epidemiology and Biostatistics, University of California San Francisco, San Francisco, CA, USA; 11 Child Life and Health, University of Edinburgh, Edinburgh, UK; 12 UK Health Security Agency, London, UK; 13 Section of Paediatric Infectious Disease, Department of Infectious Disease, Imperial College London, London, UK; 14 MRC Human Genetics Unit, Institute for Genetics and Cancer, University of Edinburgh, Edinburgh, UK; 15 School of Mathematics and Statistics, University of Glasgow, Glasgow, UK; 16 University College London, London, UK; 17 Pandemic Institute, University of Liverpool, Liverpool, UK; 18 Department of Clinical Research, London School of Hygiene and Tropical Medicine, London, UK; 136 NIHR Health Protection Research Unit, Institute of Infection, Veterinary and Ecological Sciences, Faculty of Health and Life Sciences, University of Liverpool, Liverpool, UK; 137 Respiratory Medicine, Alder Hey Children’s Hospital, Institute in The Park, University of Liverpool, Alder Hey Children’s Hospital, Liverpool, UK; 138 ISARIC Global Support Centre, Centre for Tropical Medicine and Global Health, Nuffield Department of Medicine, University of Oxford, Oxford, UK; 139 National Heart and Lung Institute, Imperial College London, London, UK; 140 Imperial College Healthcare NHS Trust London, London, UK; 141 National Infection Service, Public Health England, London, UK; 142 Centre for Inflammation Research, The Queen’s Medical Research Institute, University of Edinburgh, Edinburgh, UK; 143 Edinburgh Pathology, University of Edinburgh, Edinburgh, UK; 144 Blood Borne Virus Unit, Virus Reference Department, National Infection Service, Public Health England, London, UK; 145 Transfusion Microbiology, National Health Service Blood and Transplant, London, UK; 146 Department of Medicine, Imperial College London, London, UK; 147 Department of Pharmacology, University of Liverpool, Liverpool, UK; 148 Tropical and Infectious Disease Unit, Royal Liverpool University Hospital, Liverpool, UK; 149 Walton Centre NHS Foundation Trust, Liverpool, UK; 150 Liverpool School of Tropical Medicine, Liverpool, UK; 151 Centre for Clinical Infection and Diagnostics Research, Department of Infectious Diseases, School of Immunology and Microbial Sciences, King’s College London, London, UK; 152 Department of Infectious Diseases, Guy’s and St Thomas’ NHS Foundation Trust, London, UK; 153 Division of Infection and Immunity, University College London and Great Ormond Street Hospital, London, UK; 154 The Florey Institute for Host–Pathogen Interactions, Department of Infection, Immunity and Cardiovascular Disease, University of Sheffield, Sheffield, UK; 155 Liverpool Clinical Trials Centre, University of Liverpool, Liverpool, UK; 156 EPCC, University of Edinburgh, Edinburgh, UK; 157 Edinburgh Clinical Research Facility, University of Edinburgh, Edinburgh, UK; 158 Institute of Translational Medicine, University of Liverpool, Liverpool, UK; 159 Sheffield Teaching Hospitals, Sheffield, UK; 160 University of Liverpool, Liverpool, UK; 161 Royal Hospital for Children and Young People, Edinburgh, UK; 162 Department of Paediatric Infectious Diseases and Immunology, Royal Hospital for Children, Glasgow, UK; 163 Royal Aberdeen Children’s Hospital, Aberdeen, UK; 164 University Hospital Wishaw, North Lanarkshire, UK; 165 Crosshouse and Ayr Hospital, Kilmarnock, UK; 166 Dumfries and Galloway Royal Infirmary, Dumfries, UK; 167 Borders General Hospital, Melrose, UK; 168 Forth Valley Royal Hospital, Larbert, UK; 169 Tayside Children’s Hospital, Ninewells Hospital, NHS Tayside, Dundee, UK; 170 Ninewells Hospital, NHS Tayside, Dundee, UK; 171 Doncaster and Bassetlaw Teaching Hospitals NHS Foundation Trust, Doncaster, UK NHS Foundation Trust, Doncaster, UK; 172 Burnley General Teaching Hospital, Burnley, UK; 173 Hereford County Hospital, Hereford, UK; 174 Leighton Hospital, Leighton, UK; 175 Walsall Healthcare NHS Trust, Walsall, UK; 176 Cumberland Infirmary, Cumberland, UK; 177 Paediatric Liver, GI & Nutrition Centre and MowatLabs, King’s College Hospital, London, UK; 178 Institute of Liver Studies, King’s College London, London, UK; 179 Birmingham Women’s Children’s Hospital, Birmingham, UK; 180 St Richards’ Hospital, Chichester, UK; 181 Airedale General Hospital, Keighley, UK; 182 Hinchingbrooke Hospital, Huntingdon, UK; 183 Darlington Memorial Hospital, Darlington, UK; 184 Warrington Hospital, Kilmarnock, UK; 185 Leeds Teaching Hospitals NHS Trust, Leeds, UK; 186 Queens Hospital Burton, Burton-on-Trent, UK; 187 Queen Margaret Hospital, Dunfermline, UK; 188 Royal Victoria Infirmary, Newcastle upon Tyne, UK; 189 Poole University Hospital, Dorset, UK; 190 Bradford Royal infirmary, Bradford, UK; 191 Department of Paediatric Gastroenterology, Hepatology and Nutrition, Royal Hospital for Children, Glasgow, UK; 192 Avon and Wiltshire Mental Health Partnership NHS Trust, Bath, UK; 193 Queen Elizabeth University Hospital, Glasgow, UK; 194 University Hospital Crosshouse, Kilmarnock, UK; 195 Royal Free Hospital, London, UK; 196 Diana Princess of Wales Hospital, Grimsby, UK; 197 King’s College Hospital, London, UK; 198 Western General Hospital, Edinburgh, UK; 199 Barnsley Hospital, Barnsley, UK; 200 Bradford Teaching Hospitals NHS Foundation Trust, Bradford, UK; 201 Wye Valley NHS Trust, Hereford, UK; 202 Newcastle upon Tyne Hospitals NHS Foundation Trust, Newcastle upon Tyne, UK; 203 West Cumberland Hospital, Whitehaven, UK; 204 University Hospital of North Durham, Durham, UK; 205 Worthing Hospital, Worthing, UK

## Abstract

An outbreak of acute hepatitis of unknown aetiology in children was reported in Scotland^1^ in April 2022 and has now been identified in 35 countries^2^. Several recent studies have suggested an association with human adenovirus with this outbreak, a virus not commonly associated with hepatitis. Here we report a detailed case–control investigation and find an association between adeno-associated virus 2 (AAV2) infection and host genetics in disease susceptibility. Using next-generation sequencing, PCR with reverse transcription, serology and in situ hybridization, we detected recent infection with AAV2 in plasma and liver samples in 26 out of 32 (81%) cases of hepatitis compared with 5 out of 74 (7%) of samples from unaffected individuals. Furthermore, AAV2 was detected within ballooned hepatocytes alongside a prominent T cell infiltrate in liver biopsy samples. In keeping with a CD4^+^ T-cell-mediated immune pathology, the human leukocyte antigen (HLA) class II HLA-DRB1*04:01 allele was identified in 25 out of 27 cases (93%) compared with a background frequency of 10 out of 64 (16%; *P* = 5.49 × 10^−12^). In summary, we report an outbreak of acute paediatric hepatitis associated with AAV2 infection (most likely acquired as a co-infection with human adenovirus that is usually required as a ‘helper virus’ to support AAV2 replication) and disease susceptibility related to HLA class II status.

In April 2022, several hospitals in Scotland reported that children were presenting to medical practitioners with acute severe hepatitis of unknown aetiology^1^ (Fig. 1a). Elsewhere in the United Kingdom, 270 similar presentations were subsequently reported, for which 15 children required liver transplantation^3^. The World Health Organization (WHO) has now registered 1,010 probable cases that fulfil their definition of this illness in 35 countries^2^. Understanding the underlying cause of this new disease is a global public health imperative.

Detailed clinical investigations carried out as part of the public health response excluded common causes of acute hepatitis, including viral hepatitis, drug toxicity and autoimmune hepatitis. However, recent or active human adenovirus (HAdV) infection was identified in a high proportion of cases in Scotland, England and the United States^4–6^. This finding was notable because HAdV is not a common cause of hepatitis. An increase in HAdV diagnoses in Scotland directly preceded the out-break of unexplained hepatitis in children of a similar age (Fig. 1a,b).

SARS-CoV-2 had been circulating for 2 years and peaked several months before the increase in hepatitis cases^3^ (Fig. 1c). Human herpesvirus 6 (HHV6A and HHV6B) infections were not detected at higher levels during 2021 or 2022 (Fig. 1d).

## Research investigation

To investigate the aetiology of these cases of acute hepatitis, we recruited 32 affected children who presented to a hospital between 14 March 2022 and 20 August 2022 and met the Public Health Scotland case definition criteria for inclusion in the International Severe Acute Respiratory and Emerging Infections Consortium (ISARIC) WHO Clinical Characterization Protocol United Kingdom (CCP-UK) (ISRCTN66726260)^7^. Samples from unaffected children (control samples) were obtained from the Diagnosis and Management of Febrile Illness using RNA Personalised Molecular Signature Diagnosis (DIAMONDS) study cohort and from the NHS Greater Glasgow & Clyde (GG&C) Biorepository under appropriate ethics approval (Methods)

## Clinical presentation

The median age of affected patients was 4.1 years (interquartile range (IQR) of 2.7–5.5 years) (Table 1). All patients were of white ethnicity, and 21 out of 32 (66%) were girls. Eighteen (56%) of the children reported a subacute history 2–12 weeks before acute hepatitis, which was characterized by an initial gastroenteritis-like illness followed by intermittent vomiting, abdominal pain and fatigue. The majority (23 out of 32) had no other medical conditions. One child had previously received a liver transplant, whereas none of the other patients were immunocompromised and none had received a COVID-19 vaccination. All routine blood tests for viral hepatitis, including hepatitis A, B, C and E, acute Epstein–Barr virus (EBV), cytomegalovirus (CMV), HHV6 and HHV7, and herpes simplex virus (HSV) were negative (Supplementary Table 1). Four patients had a low titre (1:80) of anti-nuclear antibodies and 3 patients had a low titre (1:40) of anti-smooth muscle antibodies, but other markers of autoimmunity were negative (Table 1 and Supplementary Table 2).

**Fig. 1:**
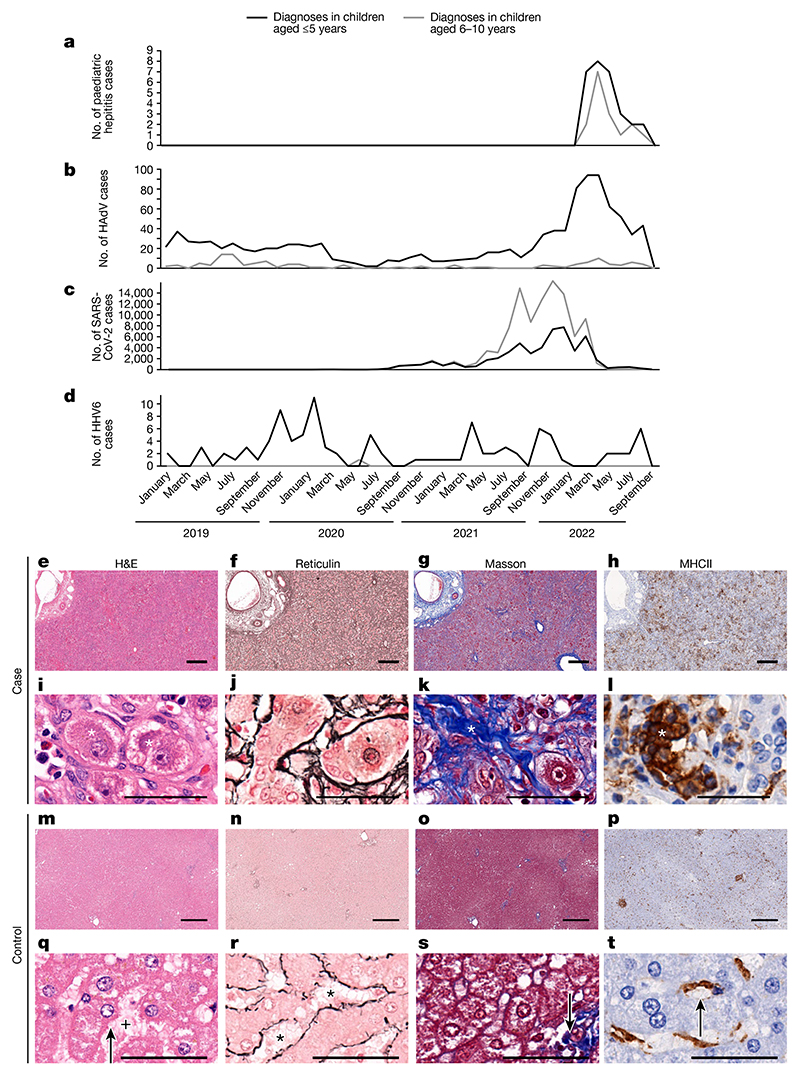
Epidemiology and histological appearance of cases of paediatric hepatitis in Scotland. **a**, The emergence of acute non-A–E hepatitis in children in March–September 2022 (ref. 3). **b**–**d**, Cases of HAdV (**b**), SARS-CoV-2 (**c**) and HHV6 (**d**) infection in children aged ≤10 years in Scotland during the period January 2019 to September 2022. **e**–**t**, Histopathology of samples from cases of non-A–E hepatitis (**e**–**l**) and from healthy liver (**m**–**t**). **e**,**i**,**m**,**q**, Serial sections of formalin-fixed and paraffin-embedded liver tissue sections (one section for each stain per patient sample) stained with haematoxylin and eosin (H&E). **f**,**j**,**n**,**r**, Reticulin staining highlighting structural organization. **g**,**k**,**o**,**s**, Masson staining highlighting collagen fibres. **h**,**l**,**p**,**t**, Staining for MHCII^+^ cells. **m**–**p**, The regular lobular structure of the liver from a healthy individual (identifier 145783) is not recognisable in **e**–**h**, which are sections collected from patient CVR35 who received a liver transplant. **h**, Immunohistochemistry showed an increase in MHCII^+^ cells in tissue samples from patient CVR35 compared with healthy liver (**l**,**t**). **i**–**l**, Higher magnification micrographs of **e**–**h** showing details of liver histopathology. **i**,**q**, For patient CVR35 (**i**), enlarged (ballooned) and vacuolated hepatocytes (marked by asterisks) are evident compared with hepatocytes in healthy liver (**q**; from individual 145783) with regular morphology (indicated by the arrow) and regular sinus (indicated by the plus symbol). **j**,**r**, For the sample from patient CVR35 (**j**), reticulin staining shows destruction of the sinus structures and irregularly arranged fibres, whereas healthy liver (**r**) shows fibres lining the sinus (indicated by asterisks). **k**,**s**, For the sample from patient CVR35 (**k**), Masson staining shows an increase in collagen fibres (in blue, indicated by the asterisk) compared with minimal staining of fibres (indicated by the arrow) in healthy liver (**s**). **l**,**t**, High magnification image showing accumulation of MHCII^+^ cells in the liver (indicated by the asterisk) of patient CVR35 (**l**), whereas healthy liver (**t**), staining is limited to Kupffer cells (indicated by the arrow). Scale bars, 50 μm (**i**–**l**,**q**–**t**) or 400 μm (**e**–**h**,**m**–**p**).

Following hospitalization, liver biopsy samples were obtained from five children. The samples showed evidence of lobular hepatitis with periportal and interface inflammation, intracellular inclusions, bile duct proliferation and ballooning of hepatocytes of varying severity (Fig. 1e–t). Mild-to-moderate fibrotic changes were noted, with no evidence of confluent fibrosis, and there was an inflammatory infiltrate that included cells expressing major histocompatibility complex class II (MHCII). Modified hepatic activity index scores (Ishak system)^8,9^ ranged from 6 to 11 (Extended Data Table 1), and the biopsy samples stained negative for complement.

Four patients required transfer to a specialist liver unit owing to significant synthetic liver dysfunction. Two of the patients were treated with steroid therapy and improved. One patient received supportive care only and spontaneously improved. The fourth patient had severe disease and required liver transplantation and was treated with cidofovir for HAdV viraemia and steroids after the liver transplant. The remaining 28 patients received supportive care only, with no antiviral or steroid treatment, and all showed gradual resolution of hepatitis over 2–3 months. There were no deaths. The median duration of hospital stay was 6 days (range of 1–68 days) (Table 1). In the patients with weakly positive autoantibodies, all had normal or normalizing transaminase levels at last follow up in the absence of treatment with an anti-inflammatory or immunosuppressant.

## Pathogen detection by sequencing

**Fig. 2:**
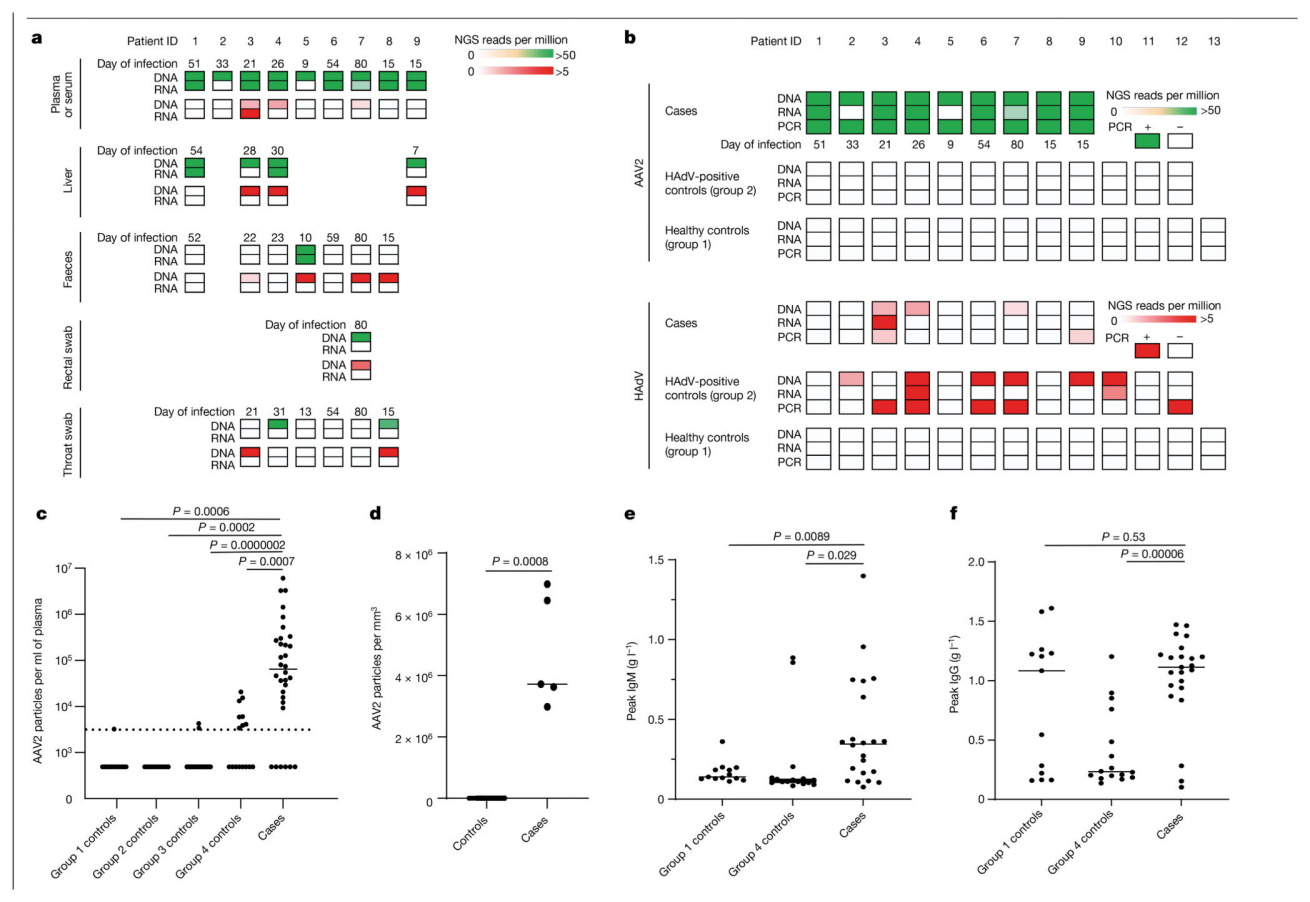
Detection of AAV2 in cases of paediatric hepatitis. **a**, Heatmap of HAdV and AAV2 reads detected in cases of hepatitis by TE sequencing. Samples obtained for routine clinical investigation (plasma, liver, faeces, rectal swab and throat swab) were retrospectively sequenced following DNA or RNA extraction. AAV2 read counts are shown from 0 to >50 reads per million in green (top rows) and HAdV read counts are shown from 0 to >5 reads per million in red (bottom rows). **b**, Heatmap of viral reads of plasma samples from cases of hepatitis and of plasma or sera samples from controls. Plasma samples from cases of hepatitis (cases), and plasma or sera samples from children with HAdV infection (group 2 controls) and from age-matched healthy children (group 1 controls) were sequenced following DNA or RNA extraction. AAV2 read counts are shown from 0 to >50 reads per million in green and HAdV read counts are shown from 0 to >5 reads per million in red. The number of days between initial symptom onset and sample are indicated. **c**, AAV2 real-time RT–qPCR of serum or plasma samples from 32 cases of hepatitis (cases) and from 74 controls in four groups: 13 in group 1 (healthy controls); 12 in group 2 (HAdV-positive controls); 33 in group 3 (hepatitis controls); and 16 in group 4 (contemporaneous controls). The detection threshold of the assay (3,200 copies per ml) is shown as a dotted line. Values are shown as a scatter plot with a median line. **d**, AAV2 real-time RT–qPCR of liver biopsy samples from 5 cases of hepatitis and from 19 controls. **e**, IgM responses determined by ELISA in 22 cases of hepatitis and in 29 controls (13 in group 3, 16 in group 4). **f**, IgG responses determined by ELISA in 22 cases of hepatitis cases and in 29 controls (13 in group 3, 16 in group 4). For **c**–**f**, statistical analysis was performed using Mann Whitney test (two-tailed), and experiments were performed in triplicate.

As the epidemiology was in keeping with the emergence of an infectious pathogen, we undertook metagenomics and target enrichment (TE) next-generation sequencing (NGS) on all available clinical samples from the first nine recruited patients. The samples included plasma (*n* = 9), liver biopsy samples (*n* = 4), throat swabs (*n* = 6), faecal samples (*n* = 7) and a rectal swab (*n* = 1), and an average of 14 million sequence reads per sample were obtained (Fig. 2a–d). The samples were obtained between 7 and 80 days after initial symptom onset. Samples from the control group were restricted to children recruited in the United Kingdom between January 2020 and April 2022. Two comparison groups were used as controls: group 1 comprised serum or plasma samples from 13 age-matched healthy children (10 boys, 3 girls; age range of 3–5 years); and group 2 comprised serum or plasma samples from 12 children (8 boys, 4 girls; age range of 1–4 years) with HAdV infection confirmed by PCR and with normal transaminase levels. The children in group 2 had been diagnosed by nasopharyngeal aspirate (*n* = 10), by nose swab (*n* = 1) or by stool (*n* = 1) as part of the routine clinical investigation process and half of the patients required critical care. The difference in age between the patients with hepatitis and the healthy children in group 1 was not significant, but some samples from group 1 were obtained earlier than samples from the cases of hepatitis (January 2020–April 2022 compared with March–April 2022, respectively) (Extended Data Table 2a). The children in group 2 were younger (median age of 1.4 years, IQR of 1.1–3.1 years, *P* < 0.001), and samples were obtained between May 2020 and December 2021 (Extended Data Table 2b). Metagenomics NGS was carried out using protocols designed to identify both RNA and DNA viruses. Semi-agnostic TE sequencing was also performed using VirCapSeq-VERT Capture probes that target the genomes of 207 taxa of viruses known to infect vertebrates.

**Extended Data Fig. 1:**
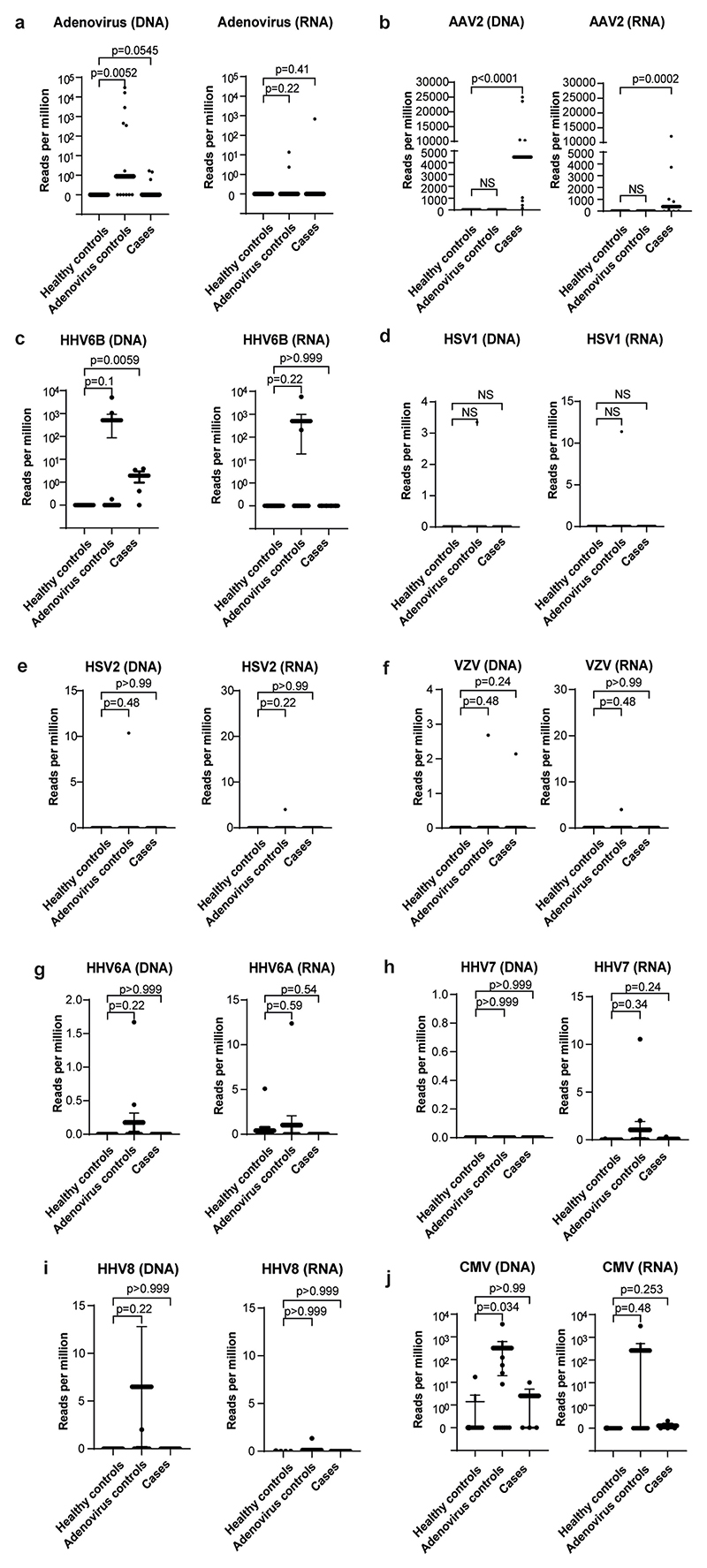
AAV2, HAdV and human herpesvirus detection by target enrichment sequencing in cases and controls. Read counts per million are plotted for a) HAdV; b) AAV2; c) HHV6B; d) HSV1; e) HSV2; f) VZV; g) HHV6A; h) HHV7; i) HHV8; and j) CMV in cases, Group 1 healthy controls and Group 2 controls (HAdV positive children with normal liver function). Statistical significance was estimated using a Mann-Whitney test (two-sided).

TE sequencing reflected the metagenomics NGS results, but with higher sensitivity, and correlated with viral loads measured by quantitative PCR with reverse transcription (RT–qPCR) (Supplementary Figs. 1 and 2). The results from both methods showed that the viral genome detected most frequently in affected patient plasma samples was AAV2 (9 out of 9 cases) (Fig. 2a, Supplementary Table 3 and Extended Data Fig. 1). AAV2 was also detected in 4 out of 4 liver biopsy samples, and in 1 out of 7 faecal samples, 1 out of 1 rectal sample and 1 out of 6 throat swab samples. At lower read counts, HAdV-F41 or HAdV-C was detected in 6 out of 9 patients, whereas HHV6B was detected in 3 out of 4 plasma samples (Extended Data Fig. 1, Supplementary Tables 4 and 5, Supplementary Data 1 and Supplementary Fig. 3). HAdV types C1, 2, 5 and 6 could not be reliably distinguished owing to low read counts. The remaining clinical samples were excluded from analysis for HHV by sequencing because murine herpesvirus 1 had been added as an extraction control during routine clinical investigation.

Read counts of AAV2 by TE sequencing were high (median of 4,478 reads per million, IQR of 774–10,498 reads per million) in all 9 out of 9 cases of hepatitis compared with 0 out of 13 in group 1 healthy controls (IQR of 0–0 reads per million, *P* < 0.001) and 0 out of 12 in group 2 controls (children with HAdV infection and normal liver function tests; IQR of 0–0 reads per million, *P* < 0.001) (Supplementary Table 5). HAdV reads were detected in 6 out of 12 HAdV-positive samples from group 2 controls (median of 0.82 reads per million, IQR of 0–1,053 reads per million) despite plasma or sera being a suboptimal sample type to detect HAdV. HAdV was detected in 3 out of 9 cases of hepatitis (median of 0 reads per million, IQR of 0–0.6 reads per million), whereas 0 out of 13 was detected in group 1 healthy controls (IQR of 0–0 reads per million, *P* = 0.055). HHV6B was also detected in 3 out of 4 cases of hepatitis compared with 0 out of 13 healthy controls (median of 1.9 reads per million, IQR of 0.3–3.5 reads per million and IQR of 0–0 reads per million, respectively, *P* = 0.006) (Supplementary Table 4). However, HHV6B read counts did not differ significantly between cases of hepatitis and group 2 controls (median of 0 reads per million, IQR of 0–0.04 reads per million, *P* = 0.16), which is in keeping with the occurrence of reactivation of HHV6B in the context of severe illness. The metagenomics and TE sequencing results from the 13 age-matched healthy control samples (group 1) revealed no evidence of AAV2, HAdV or HHV6B in plasma; however, low read counts of EBV, CMV and HHV6A were detected in a small number of samples (Supplementary Table 4). In samples from group 2 (children with HAdV infection and normal liver function tests), herpesviruses were detected in 9 out of 12 samples, including 2 out of 12 (as described above) with detectable numbers of HHV6B reads (1,050 and 5,062 reads per million), which was confirmed by PCR.

## Sequence and phylogenetic analyses

**Extended Data Fig. 2:**
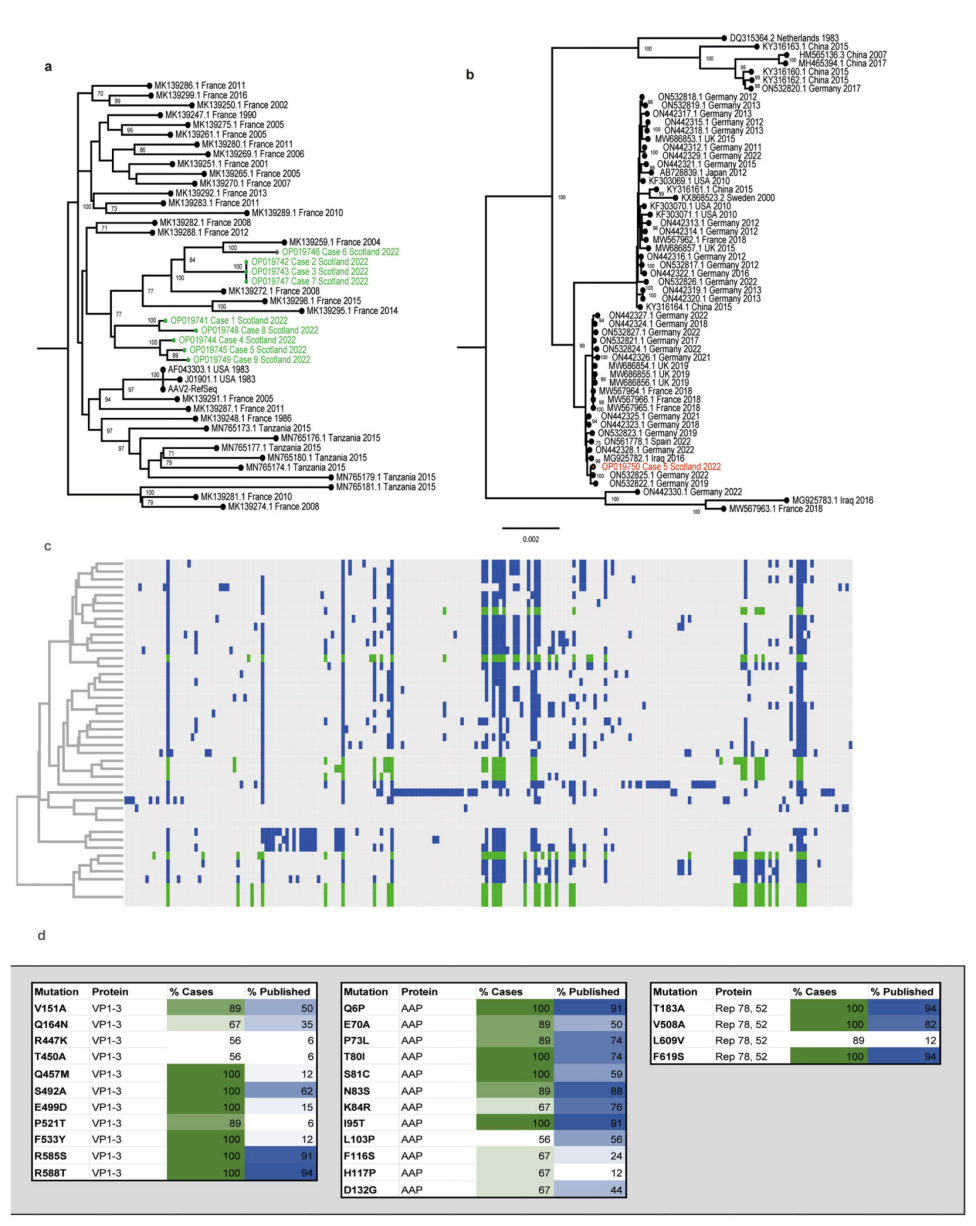
Phylogenetic and sequence analysis of AAV2 genomes. a) Maximum likelihood phylogeny of AAV2 from hepatitis cases CVR1-9. The nine AAV2 genome sequences generated from the plasma samples via target enrichment (highlighted in green) were aligned with a range of the closest AAV GenBank sequences^39^. AAV2 reference sequences are denoted by accession number, country and year of sampling b), Phylogeny of HAdV41 genome from case 5. The HAdV41 genome sequence from the faecal sample of patient 5 (red) was combined with complete genomes of HAdV41 from GenBank. Bootstrap values >70 are indicated. HAdV41 reference sequences are denoted by accession number, country and year of sampling; c), Key mutations and hierarchical clustering of AAV2 genomes. Mutations in published AAV2 sequences are highlighted in (blue) and case sequences (green); d) Mutations over-represented in hepatitis cases versus controls. Mutations in VP1-3, Rep78 and 52 and AAP are highlighted by % representation in case sequences (green) and published sequences (blue).

Near-full genomes of AAV2 were obtained from all nine patients with hepatitis (GenBank accession numbers OP019741–OP019749), and in all cases, two large open reading frames corresponding to the *rep* and *cap* genes, flanked by inverted terminal repeat regions, were identified. Seven distinct sequences of AAV2 were noted (Extended Data Fig. 2), forming a single clade, alongside four AAV2 genomes previously detected in France between 2004 and 2015. Two out of three identical sequences were known to have come from individuals from the same household, therefore these two are epidemiologically linked. The third sequence was from a sample obtained around the same time but was not known to be linked to the other cases. Sequences from the liver samples matched those detected in plasma. Several mutations within the *VP1*–*VP3* genes were noted to be over-represented in the sequences derived from patients with hepatitis when compared with reference sequences (Extended Data Fig. 2). Notably, nine of the mutations in the capsid gene that were over-represented in the cases of hepatitis (V151A, R447K, T450A, Q457M, S492A, E499D, F533Y, R585S and R588T) are associated with an AAV2 variant that has an altered phenotype. Characteristics of this variant include substantial evasion of neutralizing antibodies directed against wild-type AAV2, enhanced production yields, reduced heparin binding, increased virion stability and more localized spread in a mouse model^10^.

A full genome of HAdV-F41 was obtained from a faecal sample (GenBank accession number OP019750) and was found to be closest phylogenetically to two genomes reported from Germany in 2019 and 2022 (Extended Data Fig. 2). Contigs matching to other human pathogens, including human coronavirus NL63, rhinovirus C, enterovirus B, human parainfluenza viruses 2 and 3, norovirus, and both betaherpesvirus and gammaherpesvirus were also detected across cases, albeit not consistently. These findings were confirmed by PCR (Supplementary Table 1).

## Confirmatory PCR testing of cases of hepatitis

PCR testing for AAV2 was positive in all nine initial cases of hepatitis. Standards were used to estimate the viral loads of positive samples (Supplementary Fig. 2). All nine plasma samples tested negative by PCR for HHV6, HSV, CMV and EBV. Two out of the four liver biopsy specimens tested positive for HHV6 (cycle threshold (Ct) values of 33 and 36) (Supplementary Table 1). HAdV was detected in 3 out of 9 plasma samples, 3 out of 4 liver biopsy samples, 2 out of 6 throat swabs, 4 out of 7 faecal samples and 1 out of 1 rectal swab. The lower detection of HAdV and HHV6 by PCR compared with TE sequencing probably reflects a slightly lower sensitivity of the PCR assay. The low numbers of HAdV-positive samples detected using both assays may reflect the fact that plasma is a suboptimal sample type for HAdV detection (whole blood samples were unavailable).

## Case–control study

To investigate the presence of AAV2 and the candidate helper viruses HAdV and HHV6B in plasma samples from cases of hepatitis, we undertook a case–control study in which samples from 32 cases of hepatitis were compared with samples from the group 1 and group 2 controls described above and with samples from two additional control groups (Fig. 2a–f). Group 3 controls comprised 33 children (18 boys and 15 girls aged 2–16 years) with increased transaminase levels that had tested negative by PCR for HAdV. This group was used to test the hypothesis that reactivation of AAV2 may occur in children with severe hepatitis and may be a correlate of liver dysfunction. The children comprising group 3 were older (median age of 10.2 years, IQR of 7–13.6 years, *P* < 0.001) than the patients from the case group (Extended Data Table 2b) and 15 out of 33 had required critical care for ventilatory or cardiovascular support. Group 4 controls comprised residual plasma or serum samples from 16 children in Scotland aged 10 years and were attending hospital contemporaneously with the children with hepatitis between March and April 2022. The group 4 controls were used to determine whether AAV2 was circulating widely in children in healthcare facilities across Scotland at the time the children with hepatitis were admitted to hospital. Clinical details, including liver function were not available for this group. To ensure that the quantification of AAV2 was accurately performed, we confirmed standard curve concentrations using droplet digital PCR (Methods).

Significance differences between groups for viral loads in plasma samples were calculated using a Mann–Whitney test (two-tailed). RT–qPCR of plasma samples showed that 26 out of 32 cases of hepatitis were positive for AAV2, with a median estimated copy number of 66,100 copies per ml (IQR of 13,461– 300,277 copies per ml), a value higher than samples from all the control groups (*P* < 0.001 for all case–control comparisons). The median copy number in control groups 1–3 was below the detection limit. A median of 3,268 copies per ml (detection threshold of 3,200 copies per ml) was present in samples from control group 4, which suggested that AAV2 was circulating at low levels in children during March and April 2022 (Fig. 2c). Although five plasma samples from cases of hepatitis were positive for HAdV by PCR, and one tested positive by PCR for HHV6 DNA, these results were not significantly more common than in samples from the control group (Supplementary Fig. 3).

Next, five liver biopsy samples from cases of hepatitis were compared with 19 residual liver biopsy samples (controls) from children under 18 years old. The median AAV2 viral load was 3,721,497 copies per mm^3^ of liver (IQR of 3,308,243−6,717,616 copies per mm^3^) in cases of hepatitis compared with 64 copies per mm^3^ of liver (IQR of 20–83 copies per mm^3^) in samples from the control group (*P* < 0.001; Fig. 2d). Glyceraldehyde-3-phosphate dehydrogenase was used as a marker of extraction efficiency in all samples, and results were similar between the case and control groups. When outliers were removed, significance was retained (Supplementary Data 2, Supplementary Fig. 4).

## Longitudinal sampling

To investigate AAV2 viraemia and liver function values over time, longitudinal PCR testing was performed in 14 cases of hepatitis from whom multiple retrospective plasma samples were available (Supplementary Fig. 5). Spearman’s rank correlation coefficients for the relationships between the trajectories of viral load and alanine transaminase and bilirubin were positive for most cases. However, overall statistical significance could not be confirmed owing to the sample size.

Where samples were available, we screened for the presence of AAV2-specific IgM and IgG antibodies within samples from patients and samples from the group 1 healthy controls and group 4 contemporaneous controls (Fig. 2e,f and Supplementary Fig. 6). Anti-AAV2 IgM was detected in 15 out of 23 (65.2%) samples from cases of hepatitis, but only 1 out of 13 (7.7%) samples from group 1 healthy controls and 2 out of 16 (12.5%) samples from the group 4 contemporaneous controls from Scotland. For the samples from cases of hepatitis that tested negative for AAV2-specific IgM, samples from four patients were noted to be obtained fewer than 3 days after the onset of illness and samples from two patients were obtained more than 77 days after the onset of illness. IgG was detected in 21 out of 23 (91.3%) samples from cases of hepatitis, in 8 out of 13 (61.5%) samples from age-matched healthy controls (group 1) and in 9 out of 16 (56.3%) samples from healthy controls from Scotland (group 4). Of the two samples from patients who tested seronegative, both were obtained at early time points, probably sampled before expected seroconversion (less than 3 days after the onset of illness).

## SARS-CoV-2 infection

**Extended Data Fig. 3:**
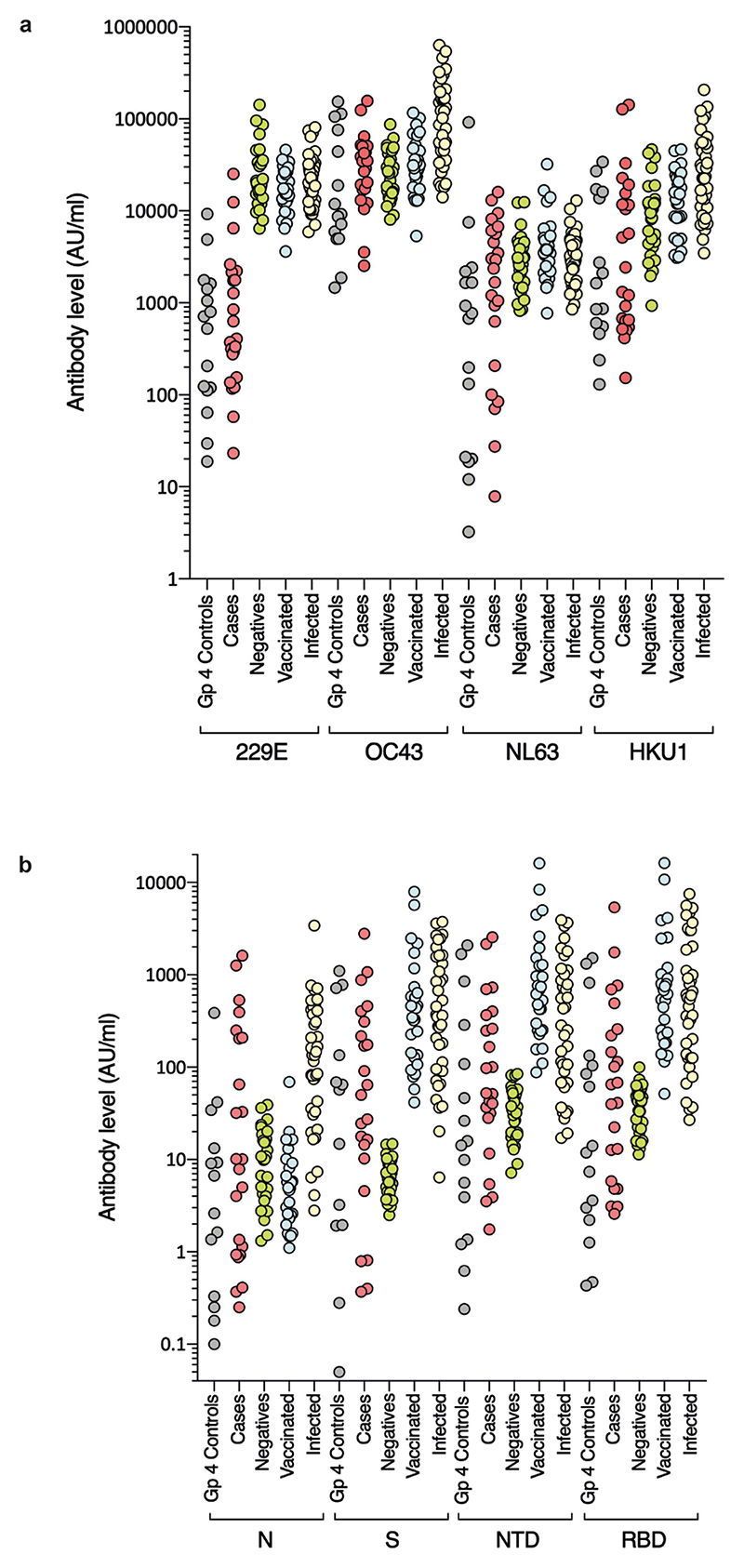
Reactivity of sera from paediatric hepatitis cases against human seasonal coronaviruses and SARS-CoV-2. Sera from the paediatric hepatitis cases were screened for reactivity against spike proteins from **a)** seasonal coronaviruses 229E, OC43, NL63 and HKU1, and **b**) SARS-CoV-2 nucleocapsid (N), spike (S), and N-terminal domain (NTD) and receptor binding domain (RBD) of S by electrochemiluminescence (MSD-ECL). Reactivity of the 23 samples (Hepatitis) was compared with 16 sera from contemporaneous control samples from children (Group 4 Controls), and three groups of sera from adults of known SARS-CoV-2 status; Negatives (never tested positive for SARS-CoV-2; n = 30), Vaccinated two doses (n = 28) and Infected (n = 39).

Routine clinical investigation detected SARS-CoV-2 nucleic acid in nasopharyngeal samples from 3 out of 31 (9.6%) children at the time of illness, 2 of whom were also seropositive. The third became infected after the onset of hepatitis. SARS-CoV-2 was not detected by PCR or by sequencing in any of the samples from cases or controls available for analysis, including liver samples. Nevertheless, to investigate the possibility that unexplained hepatitis in children might relate to a previous infection with SARS-CoV-2 or other seasonal coronaviruses, we carried out serological analysis of 23 available residual samples from cases. IgG antibody titres were quantitatively measured against the spike protein, the amino-terminal domain (NTD) and receptor binding domain (RBD) of the spike protein and the nucleocapsid of SARS-CoV-2. IgG antibody titres were also measured for human seasonal coronaviruses 229E, OC43, NL63 and HKU1. Electrochemiluminescence assays (MSD-ECL) for coronavirus-specific IgG revealed previous exposure to seasonal coronaviruses, with strong responses detected against NL63 (17 out of 23) and OC43 (21 out of 23) (Extended Data Fig. 3a). By comparison, plasma samples from 12 out of 23 children displayed high reactivity against HKU1, whereas only 3 out of 23 samples reacted strongly against 229E. Plasma samples from 11 children reacted with 2 or more SARS-CoV-2 antigens (nucleocapsid, spike protein, NTD or RBD). One of the samples reacted solely with the nucleocapsid antigen, which indicated that in total, 12 out of 23 patients displayed serological evidence of previous exposure to SARS-CoV-2 (Extended Data Fig. 3b). In summary, 12 out of 23 (52%) of the children with hepatitis displayed evidence of previous exposure to SARS-CoV-2. This level is lower than SARS-CoV-2 seroprevalence in children aged 5–11 years in Scotland between 14 March and 27 June 2022 (when Public Health Scotland enhanced surveillance for COVID-19 was discontinued), which was reported as between 59.0% (95% confidence interval (CI) of 50.6–71.2) and 72.4% (95% CI of 53.9–78.8)^11^. This result indicates that there is no direct link between COVID-19 and the outbreak of acute hepatitis studied here.

## Host genetics and HLA typing

**Extended Data Fig. 4:**
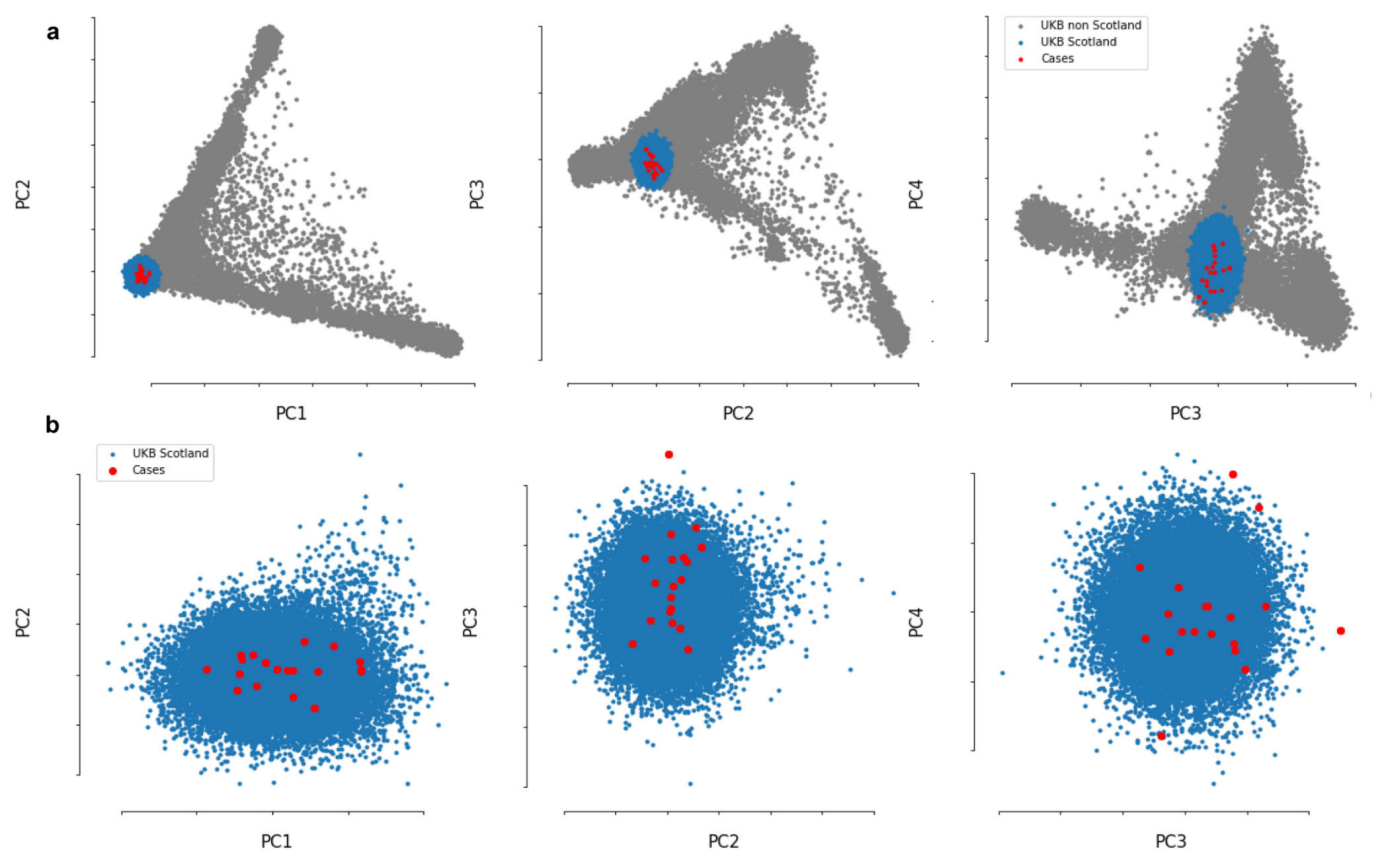
Principal component analysis (PCA) plots. PCA plots showing the first four genome-wide principal components to confirm genetic ancestry matching. **a)** Genomic PCA using full United Kingdom Biobank cohort as background population (grey), showing the subgroup of unrelated United Kingdom Biobank participants who were born in Scotland and of Caucasian ancestry (blue) and the hepatitis cases reported here (red). **b)** plots showing only the subgroup born in Scotland and of Caucasian ancestry.

We next investigated whether some children might be genetically more susceptible to non-A–E hepatitis. To that end, 27 samples from cases of hepatitis and 64 platelet apheresis samples from local donors in Scotland (controls) were genotyped using high-resolution typing for all HLA loci (*HLA-A, HLA-B, HLA-C, HLA-DRB1, HLA-DRB3, HLA-DRB4, HLA-DRB5, HLA-DQA1, HLA-DQB1, HLA-DPA1* and *HLA-DPB1*). In total, 25 out of 27 (92.6%) samples from patients with hepatitis were positive for at least one copy of the HLA-DRB1*04:01 allele compared with 10 out of 64 (15.6%) of samples from controls. The allele frequency in patients was 0.54 compared with 0.08 in controls (odds ratio (OR) of 13.7 (95% CI of 5.5–35.1), *P* = 5.49 × 10^−12^). The frequency of the HLA-DRB1*04:01 allele (based on an imputation of HLA alleles) in a control set of unrelated participants from the UK Biobank (*n* = 29,379) was 0.11 (2,942 out of 29,379 allele carriers, OR of 112.3 (95% CI of 26.6–474.5), *P* = 3.27 × 10^−23^). The frequency was also 0.11 in British/Irish North-West European individuals from the Anthony Nolan charity register^11^. To check for cryptic relatedness among patients and population stratification, we performed genome-wide microarray genotyping in 19 cases of hepatitis and excluded participants with a conservative relatedness threshold (identity-by-state > 0.4). When compared with well-matched participants from the UK Biobank (Extended Data Fig. 4), similar signals for association with disease by allele frequency (*P* = 8.96 × 10^−6^) and across the three possible biallelic genotypes at this locus (*P* = 1.2 × 10^−9^) were obtained.

In addition to the association with the *DRB1* allele, 23 out of 27 samples from patients with hepatitis were positive for HLA-DQA1*03:03 compared with 11 out of 64 samples from controls (allele frequency of 0.54 compared with 0.09, respectively, OR of 12.3 (5.1–30.7), *P* = 1.9 × 10^−11^). Moreover, 26 out of 27 samples from patients were positive for HLA-DRB4*01:03 compared with 21 out of 64 samples from controls (allele frequency of 0.67 compared with 0.17, respectively, OR of 9.4 (4.4–21.3), *P* = 1.8 × 10^−10^). Owing to strong linkage disequilibrium in this region of the genome, it is not possible to be certain which is the causal susceptibility allele.

## In situ hybridization and immune typing

**Fig. 3:**
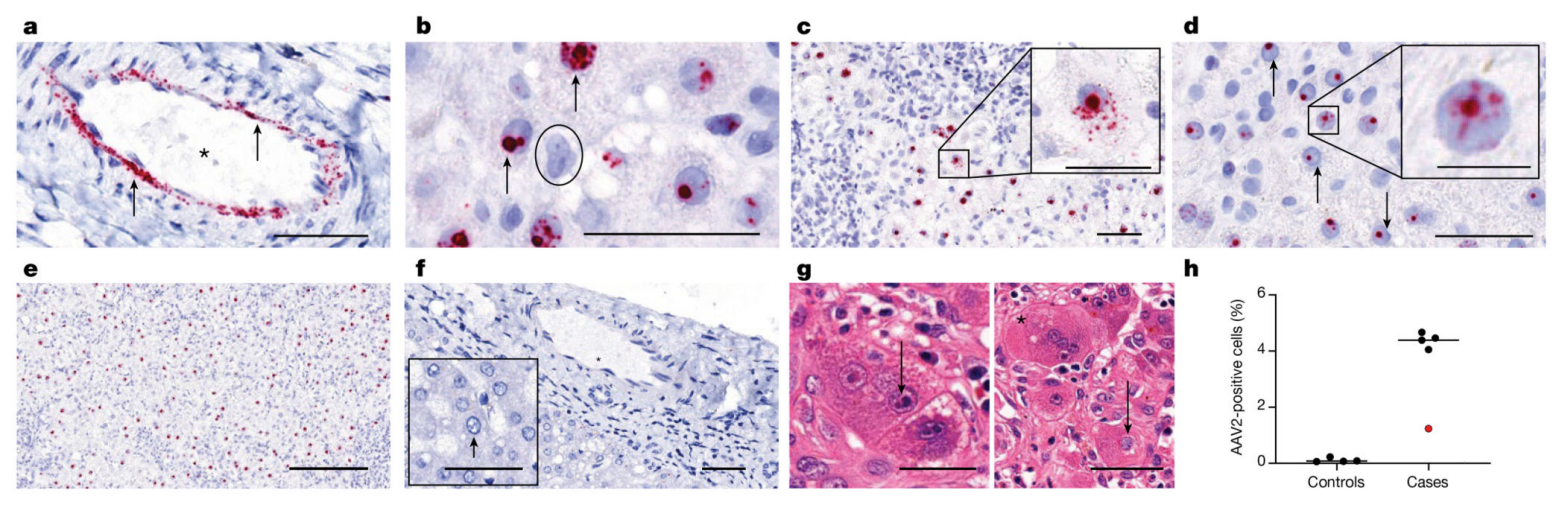
ISH of AAV2 in liver tissue. **a**–**g**, RNA ISH for the detection of AAV2 RNA in sections of formalin-fixed and paraffin-embedded liver tissues from children (one section per patient) with non A–E hepatitis. **a**, AAV2 RNA (red signal, indicated by an arrow) was detected in the endothelial cells of arteries in an explant liver section from patient CVR35. The vascular lumen is highlighted with by an asterisk. **b**, A positive AAV2 signal was detected in the nuclei of hepatocytes with vacuolated morphology from patient CVR4 (indicated by arrows) and in a negative cell (indicated by the circle). **c**,**d**, A liver section from patient CVR1 showed AAV2 RNA both in the nucleus and in the cytoplasm (**c**), whereas for patient CVR9 (**d**), AAV2 RNA was found only in the nucleus (indicated by arrows). **e**, A high percentage of hepatocytes with a positive signal for AAV2 was present predominantly in the nucleus of hepatocytes in the samples from patient CVR1. **f**, AAV2 was not detectable in liver sections from samples from healthy individuals in either the endothelial cells or hepatocytes. **g**, Samples from patient CVR35 showed inclusion bodies in hepatocytes. Left, small, dark basophilic intranuclear inclusions next to the nucleolus (indicated by arrows). Right, a large, pale basophilic, diffuse intranuclear inclusion body (suggestive of adenovirus infection; indicated by an arrow) next to a multinucleated giant cell in the liver (indicated by the asterisk). **h**, AAV2-positive cells were quantified using QuPath in biopsy samples from five patients with non-A–E hepatitis (cases) and from controls. Patient CVR35 (who received a liver transplant) is highlighted in red. Using the entire section, cells were segmented to identify the nuclei and cytoplasm, and the algorithm was tuned to detect red signals. All samples were analysed using the same algorithm. Scale bars, 25 μm (insets of **c**,**d**), 50 μm (**a**–**d**,**f**,**g**) or 200 μm (**e**).

To investigate the presence of AAV2, HAdV and HHV6 in liver biopsy samples, we carried out in situ hybridization (ISH). Liver biopsy samples of all patients were characterized by the presence of AAV2 RNA within the nuclei and cytoplasm of ballooned hepatocytes and in arterial endothelial cells, which is indicative of the presence of replicating virus (Fig. 3a–h). AAV2-positive cells were quantified at a high level in all cases using QuPath in biopsy samples from five non-A-non-E hepatitis, ranging from 1.2 to 4.7%. This level is similar to that seen in hepatitis associated with other viruses^12,13^. Consistent with low levels of HHV6B and HAdV sequence reads present in the biopsy samples from cases of hepatitis, negligible levels of viral RNA from these viruses were detected by ISH.

**Fig. 4:**
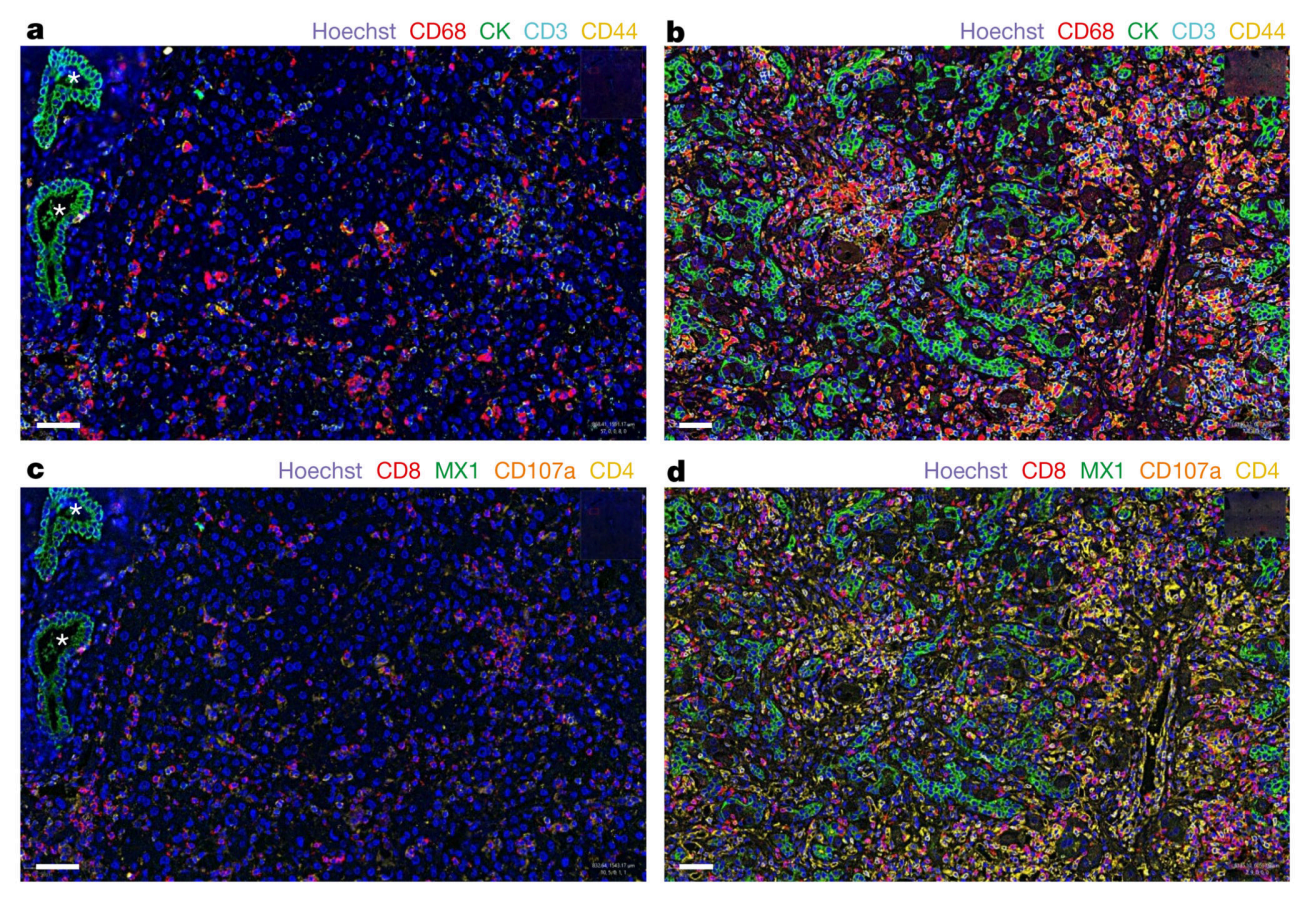
CODEX analysis of liver tissue. **a**–**d**, Images of liver tissue from patient CVR35 (**b**,**d**) and a liver sample from an unaffected individual (control; **a**,**c**) show differences in cellular composition (**c**,**d**). **a**, Regularly structured bile ducts in the liver biopsy from the control are highlighted by asterisks, and epithelial cells are stained green using cytokeratin (CK). Scattered macrophages (CD68, red), T cells (CD3, cyan) and activated T cells (CD44, yellow) are also present. **b**, By contrast, the explant liver from patient CVR35 shows prominent proliferation of epithelial cells throughout the liver tissue (green), with increased macrophages (red), T cells (cyan) and activated T cells (yellow). **c**, The control liver shows scattered cytotoxic T cells (CD8, red), CD107a-positive cells (brown) and CD4-positive cells (yellow) cells and low expression of the interferon-induced GTP-binding protein MX1 (green). **d**, High numbers of all cell types and high MX1 expression are observed in the explant liver from patient CVR35. One section of liver was stained per individual, and the entire area was manually outlined. Cells were segmented to identify the nuclei and cytoplasm, and the algorithm was tuned to detect the colour signal in the cells. All samples were analysed using the same algorithm for each stain. Scale bars, 50 μm.

To investigate the possibility of an immune-mediated pathogenesis of disease in the liver, multiplex analysis of liver samples was carried out using co-detection by indexing (CODEX) for various immune cellular markers, including CD3, CD4, CD8, PD-L1, CD107a, CD20, CD31, CD44, CD68, MX1 and PanCK (Fig. 4a–d and Supplementary Figs. 7 and 8). In the explant liver sample of patient CVR35, prominent disordered proliferation of epithelial cells throughout the liver tissue was evident, with increased numbers of CD68^+^ macrophages, activated CD4^+^ and CD8^+^ T cells and CD20^+^ B cells. High expression of the interferon-induced GTP-binding protein MX1 was also noted, which indicated that the innate immune response was activated.

## Conclusions and final statements

In this study, we reported the association of AAV2 infection and the class II HLA allele HLA-DRB1*04:01 with an outbreak of paediatric non-A–E hepatitis, with virus being detected independently by sequencing, real-time PCR and ISH. Liver biopsy tissue samples from all patients were characterized by the presence of AAV2 RNA (indicating replicating virus) within the nucleus and cytoplasm of ballooned hepatocytes and by a dense infiltrate of CD4^+^ and CD8^+^ T cells in the liver with an activated phenotype. A CD4^+^ T-helper cell-mediated immunopathological response triggered by exposure to AAV2 infection is highly probable, consistent with the markedly increased frequency of the MHC class II HLA-DRB1*04:01 allele in affected children.

AAV2 is a small non-enveloped virus with a single-stranded DNA genome of around 4,675 nucleotides in length and it belongs to the species adeno-associated dependoparvovirus A (genus *Dependoparvovirus*, family *Parvoviridae*)^14^. It was first described in 1965 and infects up to 80% of the adult population. Seroconversion occurs in early childhood following respiratory infection^15,16^. In a prospective study in the United States, the earliest seroconversion to AAV2 infection occurred in a 9-month-old child, and its seroprevalence increased from 24.2% to 38.7% in 3-year-old and 5-year-old children, respectively. This age range coincides with that of the cases in this study, which suggests that illness may be related to primary infection with AAV2 rather than its reactivation. In line with this hypothesis, anti-AAV2 IgM reactivity was observed in the majority of affected children. AAV2 relies on co-infection with a helper virus for replication, most commonly HAdV or a herpesvirus. Most clinical samples taken at presentation with hepatitis were obtained more than 20 days after initial symptom onset, which could explain the absence of a helper virus in some samples and low viral loads in positive samples. In an exploratory study using NGS, we detected two candidate helper viruses at low level in the cases of hepatitis: HAdV and HHV6B (in 6 out of 9 cases and in 3 out of 9 cases, respectively). These viruses were not confirmed to be higher in cases than controls in plasma or liver samples in our larger case–control study. HHV6B was also present in two control groups that included children with severe HAdV infection and children with hepatitis of alternative aetiology. As HHV6 can establish latency and can integrate its genome into the human chromosome, it may reactivate following concomitant illness (or immunosuppression) and may represent either an opportunistic bystander or a pathogen.

We propose that AAV2 is directly implicated in the pathology of the 2022 outbreak of non-A–E hepatitis in children, which occurred following transmission as a co-infection with HAdV or less likely due to reactivation following HAdV or HHV6 infection. Our results also support an association between HLA class II haplotype and disease susceptibility. A CD4^+^ T-cell-mediated response may direct maladaptive immunopathology mediated by T cytotoxic cells or B cells. In support of this notion, a CD8^+^ cell-mediated response directed against the AAV2 viral capsid (VP1) in association with hepatitis was reported in early trials of AAV2 when used as a vector for gene therapy^17–19^. Hepatitis remains a common phenomenon in recipients of gene therapy vectored by AAV, and this side effect is usually treated pre-emptively with steroids before and for several weeks after the gene therapy; in rare cases, AAV-mediated gene therapy has been associated with deaths from fulminant hepatic failure^20,21^. As a result of this current study, further studies are needed investigate the association between HLA status with severe illness in gene therapy recipients. Notably, we did not find features of autoimmune hepatitis (AIH), either by serology or histology, in affected children. In a study of children from Scotland with AIH^22^, the majority had evidence of seropositive disease (100% of patients with type II AIH tested positive for anti-LKM1). Furthermore, patients with AIH were older in age (median age of 11.4 years compared with 4.1 years in our cohort) and had significantly lower median alanine transaminase levels at diagnosis (444 IU per litre compared with 1,756 IU per litre). None of the AIH patients improved without treatment^22^.

An alternative explanation for our findings is that AAV2 is not directly involved in pathology and is instead a biomarker of infection with HAdV. More than half of the patients with hepatitis in our study had subacute symptoms, with a median onset of 42 days before the onset of jaundice. The opportunity to detect virus by sequencing was therefore reduced, as samples were collected after this stage of illness. Furthermore, whole blood samples might have increased the sensitivity of detection, but only serum or plasma samples were available. We consider this alternative hypothesis to be less likely because we did not detect AAV2 in a control group of children with HAdV infection who had normal liver function tests. However, HAdV41 is a common cause of diarrhoea in young children^23^, and co-infection of AAV2 with HAdV41 may explain early gastrointestinal symptoms in affected children. By contrast, although adenovirus-associated hepatitis has been previously described, particularly among immunocompromised individuals^24^, HAdV41 has not previously been associated with severe hepatitis. In the recent outbreaks of unexplained hepatitis in children, it has been inconsistently associated^4–6,25–27^.

We also investigated the possibility that the unexplained cases of hepatitis were linked to a previous illness with COVID-19. Direct SARS-CoV-2-induced liver injury is unlikely though, as few of our cases of hepatitis (3 out of 31) were positive for SARS-CoV-2 by PCR on admission to hospital, and we did not identify SARS-CoV-2 by PCR or sequencing in any of the clinical samples from cases, including liver biopsies. Furthermore, the SARS-CoV-2 seroprevalence in cases of hepatitis was lower than in the community at that time. This result is in keeping with a case–control analysis by the UK Health Security Agency^3^, who found no difference in SARS-CoV-2 PCR positivity between cases of hepatitis and children presenting to emergency departments between January and June 2022. Nevertheless, we cannot at this time fully exclude a post-COVID-19 immune-mediated phenomenon, for example, a link to HLA class II type, in susceptible children.

There are several limitations to this study. First, the presence of AAV2 in cases of hepatitis but not controls in groups 1–3 may have arisen because of seasonal variation in AAV2 transmission, as some children in the control groups were sampled earlier in the year than for cases. We included a contemporaneous control group (group 4) to address this possibility. Low viral loads of AAV2 were detected in a small number of samples from the group 4 controls, which is in keeping with the presence of the circulating virus in children at the time the cases of hepatitis occurred. Second, the presence of AAV2 in the cases of hepatitis is an association and may not represent direct aetiology, and AAV2 may be a useful biomarker of recent HAdV (or less likely HHV6B) infection. We do not consider it probable that AAV2 simply represents a marker of liver damage because it was not present in cases of severe hepatitis of alternative aetiology and, significantly, we detected AAV2 in ballooned hepatocytes by ISH. The strong association of the HLA-DRB1*04:01 allele, known to be associated with autoimmune hepatitis type 1^28^ and extra-articular manifestations of rheumatoid arthritis^29^, with the cases of hepatitis provides support for a large impact of host genetics on susceptibility. However, this analysis was affected by strong linkage disequilibrium, and larger studies are required to confirm a definitive association with this allele. The association between HLA status and the presence of an activated T cell infiltrate together with AAV2-infected cells in the liver is in keeping with a CD4^+^ cell-mediated immune pathology^30^. We consider autoimmune disease to be less likely of a cause of the cases of hepatitis studied here because of the absence of autoantibodies and the absence of typical histology in liver specimens. It is also plausible that simultaneous HAdV infection with a co-infecting or reactivated AAV2 infection has resulted, for a proportion of children who are more susceptible (owing to the HLA class II allele HLA-DRB1*04:01), in a more severe outcome than might typically be expected for these commonly circulating viruses. Peptide mapping experiments are recommended in future studies to investigate the nature of the HLA class II-restricted T cell response.

The 2022 outbreak of AAV2-associated paediatric hepatitis that we described in this study may have arisen because of changes in exposure patterns to AAV2, HAdV and HHV6B as an indirect consequence of the COVID-19 pandemic. The circulation of common human viruses was interrupted in 2020 by the implementation of non-pharmaceutical interventions, including physical distancing and travel restrictions, instituted to mitigate SARS-CoV-2 transmission. Once restrictions were lifted, genetically susceptible children may have had a higher chance of being exposed to both HAdV and AAV2 for the first time, creating a synchronized wave of severe disease. Larger case–control studies are needed to confirm the role of AAV2 and HLA status in the aetiology of unexplained non-A–E paediatric hepatitis. Retrospective testing of samples from sporadic cases of unexplained hepatitis in children is also needed.

## Methods

### ISARIC CCP-UK recruitment, Biorepository and DIAMONDS studies

Ethics approval for the ISARIC CCP-UK study was given by the South Central–Oxford C Research Ethics Committee in England (13/SC/0149), the Scotland A Research Ethics Committee (20/SS/0028) and the WHO Ethics Review Committee (RPC571 and RPC572). Thirty-two children aged <16 years were prospectively recruited by written informed consent (parent or guardian) from the ISARIC WHO CCP-UK cohort admitted to hospital with increased transaminase levels (defined as alanine transaminase levels of >400 IU per litre and/or aspartate aminotransferase levels of >400 IU per litre) that was not due to viral hepatitis A–E, AIH or poisoning. Nine patients had available clinical samples for further investigation. Three additional patients had HLA typing performed, but samples were not available for further analysis. Samples for the control groups were obtained from children (aged <16 years) recruited to the DIAMONDS study, an ongoing multi-country study that aims to develop a molecular diagnostic test for the rapid diagnosis of severe infection and inflammatory diseases using personalized gene signatures (ISRCTN12394803). Ethics approval was given by the London-Dulwich Research Ethics Committee (20/HRA/1714). Controls included healthy individuals (*n* = 13; group 1), children with PCR-confirmed adenoviral infection with normal transaminase levels (*n* = 12; group 2), and children with increased transaminase levels without adenoviral infection (*n* = 33; group 3), recruited between 19 May 2020 to 8 January 2022. Surplus plasma samples from individuals in Scotland (aged <10 years; March to April 2022; group 4) and liver biopsy control samples (from individuals aged <18 years; January 2021 to July 2022) from the Diagnostic Pathology/Blood Sciences archive were obtained with NHS GG&C Biorepository approval (application no. 717; REC 22/WS/0020). Samples from adults that had tested negative by PCR for SARS-CoV-2 were used as an additional group for serological analysis of coronaviruses as a negative control group, also with NHS GG&C Biorepository approval. These adult samples were used without consent on the basis of Human Tissue Act legislation on consent exemption.

### Viral PCR

RNA extraction was carried out using the protocol from Biomerieux Easymag. In total, 300 μl of plasma or sera was extracted and eluted into 80 μl of water.

AAV2 RT–qPCR was performed to detect a 62 bp amplicon of the AAV2 inverted terminal repeat region (ITR) as previously described^31^ using the forward ITR primer (5′-GGAACCCCTAGTGATGGAGTT-3′) and the reverse ITR primer (5′-CGGCCTCAGTGAGCGA-3′). The AAV2 ITR hydrolysis probe was labelled with fluorescein (6FAM) and quenched with Black Hole quencher (BHQ) 5′-[6FAM]-CA CTCCCTCTCTGCGCGCTCG-[BHQ1]3′). AAV2 primers and probe were synthesized by Merck Life Sciences. RT–qPCR analysis was performed using an ABI7500 Fast Real-Time PCR system (Applied Biosystems). A LUNA Universal One-Step RT PCR kit (New England Biolabs) was used for the amplification and detection of the AAV2 ITR target. RT–qPCR assays were performed in a 20 μl volume reaction (Luna Universal One-Step reaction mix, Luna WarmStart RT enzyme mix, 400 nM forward and reverse primers, 200 nM AAV2 ITR probe and 1–2.5 μl of template DNA) as per the manufacturer’s instructions. To quantify the number of copies, serial dilutions of plasmid containing the 62 bp ITR product were used to generate a standard curve, which was then used to interpolate the copy number of AAV2 copies in the samples. Wells with no template were used as negative controls. RT–qPCR reactions were performed in triplicate. The RT–qPCR program consisted of an initial reverse transcription step at 55 °C for 10 min, an initial denaturation step at 95 °C for 1 min followed by 45 cycles of 95 °C denaturation for 10 s and extension at 58 °C for 1 min. A qPCR detection limit between 31 and 32 cycles was calculated as the threshold Ct value at the last dilution of DNA standards that were within the linear range. A PCR result was considered positive if all three reactions tested positive at ≤31 cycles. Digital droplet PCR was performed according to the manufacturer’s instructions using the digital droplet PCR supermix for probes (no dUTP) (Bio-Rad, 1863023) and analysed using a QX200 Droplet Digital PCR system (Bio-Rad, 1864001).

The West of Scotland Specialist Virology Centre, NHS Greater Glasgow and Clyde, conducted diagnostic real-time RT–PCR to detect HAdV, SARS-CoV-2-positive samples and other viral pathogens associated with hepatitis (for example, hepatitis A–E) following nucleic acid extraction utilizing the NucliSENS easyMAG and Roche MG96 platforms. HHV6 (ref. 32) and HAdV41 (ref. 33) were tested by qPCR as previously described using Invitrogen platinum qPCR mix (11730-025) and Quanta Biosciences qPCR mix mastermix (733-1273), respectively, on an ABI7500 system and amplified for 40 cycles. A 6 μl extract was amplified in a total reaction volume of 15 μl.

### Measurement of antibody response to coronaviruses by electrochemiluminescence

IgG antibody titres were quantitatively measured against the spike protein, the NTD, the RBD or nucleocapsid of SARS-CoV-2, and against the spike glycoproteins of human seasonal coronaviruses 229E, OC43, NL63 and HKU1 using MSD V-PLEX COVID-19 Coronavirus Panel 2 (K15369) and Respiratory Panel 1 (K15365) kits. Multiplex meso scale discovery electrochemiluminescence (MSD-ECL) assays were performed according to manufacturer’s instructions. Samples were diluted 1:5,000 in diluent and added to the plates along with serially diluted reference standard (calibrator) and serology controls 1.1, 1.2 and 1.3. Plates were read using a MESO Sector S 600 plate reader. Data were generated using Methodological Mind software and analysed using MSD Discovery Workbench (v.4.0). Results are expressed as MSD arbitrary units per ml (AU ml^–1^). Adult negative and positive pools gave the following values: negative pool: spike, 56.6 AU ml^–1^; NTD, 119.4 AU ml^–1^; RBD, 110.5 AU ml^–1^; and nucleocapsid, 20.7 AU ml^–1^; SARS-CoV-2-positive pool: spike, 1,331.1 AU ml^–1^; NTD, 1,545.2 AU ml^–1^; RBD, 1,156.4 AU ml^–1^; and nucleocapsid, 1,549.0 AU ml^–1^. In the same assay, NIBSC 20/130 reference serum was used and the following values obtained: spike, 547.7 AU ml^–1;^ NTD, 538.8 AU ml^–1^; RBD, 536.9 AU ml^–1^; and nucleocapsid 1,840.2 AU ml^–1^.

### Metagenomics sequencing

Full protocols on the detection of RNA and DNA viruses using metagenomics NGS and TE sequencing methods can be found in refs. 32,34.

In summary, residual nucleic acid from 27 samples from cases with hepatitis (from 9 patients with a combination of plasma, liver, faeces, rectal, and throat and nose samples), 12 samples from HAdV-positive individuals and 13 samples from healthy individuals (control samples were either plasma or sera) underwent metagenomics NGS sequencing at the MRC-University of Glasgow Centre for Virus Research Genomics facility. In brief, each nucleic acid sample was split into two library preparations to improve the chances of detecting RNA and DNA viruses. The protocol used to improve detection of RNA viruses included treatment with DNaseI (Ambion DNase I, ThermoFisher), ribosomal depletion (Ribo-Zero Plus rRNA Depletion Kit, Illumina), except for plasma samples, reverse transcription (SuperScript III, Invitrogen) and double-strand DNA synthesis (NEBNext Ultra II Non-Directional RNA Second Strand Synthesis Module, NEB). The protocol used to detect DNA viruses included partial removal of host DNA (NEBNext Microbiome DNA Enrichment Kit, NEB). Following this, both sets of samples were used to prepare libraries using a KAPA LTP kit (Roche) with unique dual indices (NEBNext Multiplex oligos for Illumina, NEB). The resulting libraries were pooled in equimolar amounts and sequenced using a NextSeq500 (Illumina) to obtain paired-end reads using 150 × 150 cycles.

### TE sequencing

Following the library preparation step described above, DNA-derived and RNA-derived libraries were pooled separately and were incubated with VirCapSeq-VERT Capture Panel probes (Roche) following the manufacturer’s guidelines. The Roche VirCapSeq-VERT Capture Panel covers the genomes of 207 taxa of viruses known to infect vertebrates (including humans). Enriched DNA-derived and RNA-derived libraries were further amplified using 14 PCR cycles, then pooled and sequenced using a NextSeq500 (Illumina) to obtain paired-end reads using 150 × 150 cycles.

### Bioinformatics analysis

Reads for each sample were first quality checked. Illumina adapters were trimmed using Trim Galore (https://github.com/FelixKrueger/TrimGalore) and then mapped to the human genome using BWA-MEM (https://github.com/lh3/bwa). Only reads that did not map to the human genome were used for metagenomics analyses. Reads per million were calculated as the number of viral reads per million reads sequenced to normalize for variation in sample sequencing depth. Non-human reads were then de novo assembled using MetaSPAdes (https://github.com/ablab/spades) to generate contigs for each sample. Contigs were compared against a protein database of all NCBI RefSeq organisms (including virus, bacteria and eukaryotes) with BLASTX using DIAMOND (https://github.com/bbuchfink/diamond). In addition, non-human reads for each sample were aligned to a small panel of HAdV NCBI RefSeq genomes (HAdV-A, HAdV-B1, HAdV-B2, HAdV-C, HAdV-D, HAdV-E, HAdV-F, HAdV-1, HAdV-2, HAdV-5, HAdV-7, HAdV-35, HAdV-54 and HAdV-F41).

The nine AAV2 near-complete genome contigs from the plasma samples were assembled and compared with sequences in GenBank using BLASTN (nucleotide database). Each of these AAV2 genomes had numerous close hits (exhibiting >95% similarity across 95% of the genome) with various existing AAV2 sequences; those most closely related were reported in a previous publication^35^. All linear complete AAV2 genomes returned from BLAST against the GenBank nucleotide database with a query coverage of >75% were selected and combined with the AAV sequences de novo assembled here and aligned using MAFFT. The terminal ends of this alignment were trimmed off, and IQ-TREE 2 was used (TIM+F+R3 model) to infer a phylogenetic tree. For the single HAdV41 genome de novo assembled, all available HAdV41 complete genomes were downloaded from GenBank, aligned with MAFFT and IQ-TREE2 was used (K2P+R2 model) to infer a phylogenetic tree.

### Anti-AAV2 ELISA

AAV2 pAAV-CAG-tdTomato viral preparation (codon diversified) was a gift from E. Boyden (Addgene viral preparation number 59462-AAV2; http://n2t.net/addgene:59462; RRID:Addgene_59462).

AAV2 particles, obtained from Addgene (59462-AAV2) were diluted in PBS and used to coat a Immulon 2HB 96-well flat bottom plate (Immuno-Chemistry Technologies) at a concentration of 1 × 10^8^ particles per well. The plates were incubated on an orbital shaker overnight at 4 °C. Plates were then blocked with PBS-T (PBS with 0.1% Tween-20) containing 5% BSA for 1 h before the addition of samples. The plates were washed five times in PBS-T before serum samples, diluted 1:50 in PBS, were added in triplicate. A mouse anti-AAV2 (A20, Progen) was used as a positive control at a concentration of 1:50. Samples were incubated at room temperature on an orbital shaker for 90 min before washing five times in PBS-T and adding either anti-human IgM or anti-human IgG (Merck, A9794 and A1543, respectively) diluted 1:10,000. Goat anti-mouse IgG (Merck, A2429) was used as the secondary for the anti-AAV2 A20 positive control. The plates were incubated for 1 h before washing five times with PBS-T then 100 μl of alkaline phosphatase yellow (Merck, P7998) was added and incubated for 15 min before stopping the reaction with 3 M NaOH and the absorbance measured at 405 nm.

### Immunohistochemistry, ISH and special staining

Formalin-fixed and paraffin-wax-embedded liver samples were cut at around 3 μm thickness and mounted on glass slides. A reticulin (1936) and Masson trichrome (1929) special staining method (Gordon and Sweets method (1936)) was performed. Antibodies used for immuno-histochemistry are listed in Supplementary Table 6.

Detection of viral nucleic acids, ubiquitin and DapB-specific RNA (Advanced Cell Diagnostics, AAV2 (1195791), HHV6 (144565), adenovirus 41 (1192351), ubiquitin (310041) and DapB (310043)) was performed following the manufacturer’s protocol with pretreatment with simmering in target solution (30 min) and additional proteinase K (30 min) treatment. A haematoxylin counterstain was performed, and slides were mounted with Vectamount mounting medium (H-500, Vector Laboratories) and scanned using a bright-field slide scanner (Leica, Aperio Versa 8).

### Liver histopathology grading

Liver scoring was performed as previously described^8,9^.

### Quantification of immune cells

After scanning of the whole slide, liver tissue was outlined and the number of positively stained cells (DAB signal for immunohistochemistry or Fast Red signal for ISH) was assessed using software-assisted image analysis (QuPath, v.0.3.2)^36^. For each marker, the cell detection algorithm was tuned, and data were plotted using GraphPad Prism (v.9.4.1).

### Spatial analysis (CODEX Phenocycler)

Formalin fixed, paraffin-wax-embedded liver samples (patient 228742A and patient 145808) were sectioned at 2–4 μm thickness on 22 × 22 mm glass coverslips (Akoya Biosciences, 7000005) coated in 0.1% poly-l-lysine (Sigma-Aldrich, P8920). Antigen retrieval was performed by pressure cooking with citrate buffer at pH 6. Carrier-free, pre-conjugated antibodies were purchased directly from Akoya Biosciences or purchased from other suppliers in preparation for custom conjugation. If carrier-free antibodies were not available, alternatives were purchased and purified using a Pierce antibody cleanup kit (44600, ThermoFisher). Antibodies were custom conjugated to a unique oligonucleotide barcode according to the manufacturer’s instructions using an antibody conjugation kit (7000009, Akoya Biosciences) and stored at 4 °C for at least 48 h before use. Conjugated antibodies were stored at 4 °C.

Coverslips with tissue were rehydrated in an alcohol series and washed in distilled water before performing heat-induced antigen retrieval in a pressure cooker with citrate buffer (pH 6). Glass coverslips were then moved progressively between wells of a 6-well plate containing components of the CODEX staining kit (Akoya Biosciences, 7000008). This included 2 wells of hydration buffer (2 min each), 1 well of staining buffer (20 min), and then staining with 190 μl of an 11-marker antibody panel (Supplementary Table 7). Tissue sections of both samples were treated in the same way on the same day and were incubated with antibodies for 3 h at room temperature simultaneously. Following staining, tissue was incubated twice in staining buffer (2 min each) and transferred to a post-staining fixation solution made from a 1:10 ratio of paraformaldehyde to storage buffer for 10 min. Tissue samples were then washed 3 times in 1× PBS (14190-094, Gibco), incubated in ice-cold methanol (M/4000/PC17, Fisher Scientific) for 5 min on ice, and again washed 3 times in PBS. Tissue sections were fixed in a fixative solution for 20 min, washed 3× in PBS and stored in storage buffer until image acquisition.

Image acquisition was achieved using a Keyence BZ-X710 microscope equipped with 4 fluorescent channels (1 nuclear stain, 3 for antibody visualization). In a 96-well plate (Akoya Biosciences, 7000006), a maximum of three oligonucleotide reporters were used per well (cycle) (5 μl each) and added to between 235 μl and 245 μl reporter stock solution that was made according to the manufacturer’s instructions. Plates were sealed with aluminium film (Akoya Biosciences, 7000007) and stored at 4 °C until use. Pictures were captured using QuPath (v.0.3.2)^36^.

### Host genetics and HLA typing

High-resolution typing for all HLA loci (*HLA-A, HLA-B, HLA-C, HLA-DRB1, HLA-DRB3, HLA-DRB4, HLA-DRB5, HLA-DQA1, HLA-DQB1, HLA-DPA1* and *HLA-DPB1*) was performed using an AllType FASTplex NGS assay (One Lambda) run on an Illumina Mi-Seq platform. HLA typing was undertaken on 27 ISARIC participants who provided consent. One patient was omitted from analysis as they were a sibling of another case. HLA types from 64 Scottish National Blood Transfusion Service apheresis platelet donors, self-identified as white British (*n* = 15) or white Scottish (*n* = 49) were used as control samples for comparison with patient HLA allele frequencies. Genotyping was performed using Illumina Global Screening Array v.3.0 + multi-disease beadchips (GSAMD-24v3-0-EA) and Infinium chemistry. This consists of three steps: (1) whole genome amplification; (2) fragmentation followed by hybridization; and (3) single-base extension and staining. Arrays were imaged on an Illumina iScan platform, and genotypes were automatically called using GenomeStudio Analysis software (v.2.0.3), GSAMD-24v3-0-EA_20034606_A1.bpm manifest and a cluster file provided by the manufacturer.

Given the small sample size, it was not possible to implement quality control processes using GenomeStudio and the manufacturer’s published recommendations. As genotyping was conducted using the same genotyping array used for the GenOMICC study, variants that passed quality control for the GenOMICC study were retained as previously described^37^. After further excluding variants with call rates of <95%, a total of 478,692 variants were used for downstream analysis.

### Kinship and population structure

To identify close relatives up to third degree, King 2.1 was used, which confirmed the presence of a pair of siblings with no further close relatives identified. Genotypes of 19 patients were combined with imputed genotypes of a subset of unrelated participants from the UK Biobank, which was obtained by removing one individual in each pair with estimated kinship larger than 0.0442. The resulting genotypes were filtered to exclude variants with a mean allele frequency of <5%, a genotype missingness rate of <1.5% and Hardy–Weinberg equilibrium of *P* < 10^−50^. Principal component analysis was conducted with gcta 1.955 in the set of unrelated individuals with pruned single nucleotide polymorphisms using a window of 1,000 markers, a step size of 50 markers and a *r*^2^ threshold of 0.01. Analyses were performed once including all UK Biobank participants and once including only UK Biobank participants who were born in Scotland (UK Biobank data-field 1647) and of Caucasian genetic ancestry (UK Biobank data-field 22006).

### Statistics

Differences between cases and control groups were tested using Fisher’s exact test for categorical variables and Mann–Whitney (two tailed) for continuous variables using R studio (v.1.2.5033), R (v.4.1.2) and GraphPad (v.9.0.0).

For coronavirus serology experiments, comparisons were carried out using one-way analysis of variance (ANOVA) and Tukey’s multiple comparison test, carried out in GraphPad (v.8.4.3).

HLA analysis used the Bridging ImmunoGenomic Data-Analysis Workflow Gaps (BIGDAWG) R package to derive OR and corrected *P* values for individual HLA alleles^38^. The Bonferroni-corrected *P* value significance threshold, adjusted for multiple comparisons (168 HLA alleles), was *P* < 3.0 × 10^−4^.

### Figures

Figures were prepared using Microsoft Office Excel 2010, Microsoft Office PowerPoint 2010 and Adobe Illustrator 2022.

### Reporting summary

Further information on research design is available in the Nature Portfolio Reporting Summary linked to this article.

## Extended Data

**Extended Data Table 1:** Modified hepatic activity index scores

	CVR1	CVR3	CVR4	CVR9	CVR35
**Modified Ishak score**	6/18	10/18	10/18	8/18	11/18
**Portal inflammation**	2/4	4/4	4/4	2/4	2/4
**Interface hepatitis**	2/4	4/4	4/4	4/4	4/4
**Confluent necrosis**	0/6	0/6	0/6	0/6	1/6
**Lobular inflammation**	2/4	2/4	2/4	2/4	4/4

**Extended Data Table 2:** Characteristics of cases and controls a) used in metagenomic and target enrichment analysis b) used in PCR analysis

a									
	Cases - metagenomic & TE sequencing analysis (n=9)	Controls							
		Group 1 DIAMONDS Healthy (n=13)		P value*	Group 2 DIAMONDS HAdV infection with normal transaminases (N=12)		P value*		
Sex - male	4 (44.4)	10(76.9)		0.19	7 (58.3)		0.67		
Age (years)	3.9 (3.4-5.1)	4.1 (3.6-4.8)		0.87	1.4(1.1-3.1)		0.0006		
Recruitment period	14 March – 20 April 2022	6 Nov 2020 - 6 Jul 2021		-	22 May 2020 - 22 Dec 2021		-		
**b**									
	**Cases – PCR** (n=32)	**Controls**							
		Group 1 DIAMONDS Healthy (n=13)	P value*	Group 2 DIAMONDS HAdV infection with normal transaminases (N=12)	P value*	Group 3 DIAMONDS Elevated transaminases with no HAdV infection (n=33)	P value*	Group 4 Scottish hospitalised controls (n=16)	P value*
Sex - male	11 (34.4)	10(76.9)	0.019	7 (58.3)	0.18	17(51.5)	0.21	-	-
Age (years) †	4.1 (2.7-5.5)	4.1 (3.6-4.8)	0.95	1.4(1.1-3.1)	0.0002	10.2(7-13.6)	<0.0001	-	-
Recruitment period	14 March – 20 August 2022	6 Nov 2020 - 6 Jul 2021	-	22 May 2020 - 22 Dec 2021	-	9 Sep 2020 - 8 Jan 2022	-	12 March - 4 April 2022	-

* Fisher’s Exact or chi-squared test for categorical and Mann-Whitney (two-sided) test for continuous variables.

† Age and sex of Group 4 controls unavailable.

## Supplementary Material

Supplementary

Supplement Reporting Summary

## Figures and Tables

**Table 1:** Demographic and clinical characteristics of the 32 patients with unexplained hepatitis

Demographics	Results
Age (years)^a^	4.1 (2.7–5.5, 0.9–10.6)
Sex (girls)^b^	20 (63%)
Co-morbidity^b^	9 (28%)^c^
**Biochemistry**	
Peak bilirubin^a^ (μmoll^−1^)	82 (36–160, 3–387)
Peak alanine transaminase^a^ (Ul^−1^)	1,757 (708–2,763, 333–5,417)
Peak aspartate transaminase^a^ (U l^−1^)	2,048 (833–3,408, 424–6,908)
Peak y-glutamyltransferase^a^ (U l^−1^)	124 (91–162, 18–720)
Peak international normalized ratio^a^	1.2 (1.1–1.4, 1.0–2.9)
Peak C-reactive protein^a^ (mgl^−1^)	5 (3–11, 1–117)
Caeruloplasmin^a^ (*n*=24) (g l^−1^)	0.36 (0.33–0.39, 0.22–0.52)
**Key autoimmune parameters**	
IgG^a^ (gl^−1^)	11.8 (9.9–14.3, 1.5–21.0)
Coeliac screen (TTG antibody) (*n*=26)	26 normal range
Anti-mitochondrial antibody	32 negative
Anti-smooth muscle antibody	29 negative, 3 low positive (1:40)^c^
Anti-liver kidney microsomal 1 antibody	32 negative
Anti-nuclear antibody	28 negative, 4 weak positive 1:80 titre^c^
**Clinical presentation**	
Symptoms at presentation^b^	
• Vomiting	22 (69%)
• Jaundice	21 (66%)
• Poor appetite	12 (38%)
• Lethargy or fatigue	10 (31%)
• Abdominal pain	10 (31%)
• Diarrhoea	4 (13%)
Subacute symptoms for ≥14 days before presentation (*n* = 32)	18 (56%)
Subacute symptoms reported (*n* = 18)	
• Intermittent vomiting	15 (83%)
• Initial gastroenteritis-like illness	12 (67%)
• Abdominal pain	9 (50%)
• Lethargy or fatigue	7 (39%)
• Poor appetite	6 (33%)
• Weight loss	6 (33%)
Approximate duration of subacute symptoms before presentation^a,d^	42 (27–52, 14–85) days
Length of hospital stay^a,e^	6 (4–10, 1–68) days
Required transfer to tertiary liver unit	4 (12.5%)
Required liver transplant	1 (3%)

a Median (IQR, range).

b Number (%) denominator = 32 unless otherwise specified.

c See Supplementary Information for additional clinical details.

d *n* = 16 patients with data available.

e *n* = 30, one patient was a long-term in-patient for an unrelated condition, one patient was managed as an outpatient.

## Data Availability

Datasets generated in the current study are appended as supplementary tables. Data, protocols and all documentation regarding this analysis may be made available to academic researchers after authorization from the independent data access and sharing committee. Clinical data and analysis scripts are available on request to the Independent Data Management and Access Committee at https://isaric4c.net/sample_access. Restrictions apply to the availability of identifiable clinical data. Owing to the relatively small number of cases, de-aggregation of data is potentially disclosive, as is the patient-level line list data. Therefore, a formal data-sharing agreement is required for data access. The Independent Data and Material Access Committee considers requests as they arrive; most responses are made within 28 days. Use of clinical samples are also restricted under ethical approvals obtained for their use. Genome sequences are available at GenBank with accession numbers OP019741–OP019749 for AAV2 and OP019750 for HAdV-F41. Source data are provided with this paper.

## Code availability

Freely available bioinformatics and statistical software were used, see links in the Methods section.
