# Supplementary material for: Adeno-associated virus 2 infection in children with non-A–E hepatitis
Source: Nature. Author manuscript; Available in PMC 2025 May 12. (PMC7617659; doi:10.1038/s41586-023-05948-2)
Supplement: Supplementary [file EMS203901-supplement-Supplementary_.pdf]

nature portfolio

https://doi.org/10.1038/s41586-023-05948-2

Supplementary information

Adeno-associated virus 2 infection in children with non-A–E hepatitis

In the format provided by the authors and unedited

Adeno-associated virus 2 infection in children with non-A-E hepatitis

Supplementary Information

Contents

1. SUPPLEMENTARY CLINICAL INFORMATION 3

1.1. Approach to clinical investigation and diagnosis 3

1.2. Comorbidities and prior medication 3

1.3. Autoantibody testing 4

1.4. Ethical approvals 4

2. SUPPLEMENTARY TABLES 5

Supplementary Table 1. Clinical virology results of the 32 non-A-E hepatitis cases in Scottish children 5

Supplementary Table 2. Non-virological clinical results of the 32 non-A-E hepatitis cases in Scottish children 9

Supplementary Table 3. Read counts per million of AAV2 and HAdV in cases following target enrichment sequencing 11

Supplementary Table 4. Read counts per million of HHV in case and control sera following target enrichment sequencing 12

Supplementary Table 5. Read counts per million of AAV2 and HAdV in case and control sera following target enrichment sequencing 13

Supplementary Table 6. Antibodies used for immunohistochemistry 13

Supplementary Table 7. Panel for Codex analysis 14

3. SUPPLEMENTARY FIGURES 15

Supplementary Figure 1. AAV2 NGS read counts plotted against RT-PCR cycle Threshold (Ct) and estimated viral loads 15

Supplementary Figure 2. Cycle threshold (Ct) values and estimated copy number for AAV2 real-time PCR 16

Supplementary Figure 3. Cycle threshold (Ct) values for cases and controls for HHV6 and HAdV PCR 17

Supplementary Figure 4. GAPDH values for liver biopsy cases and controls 18

Supplementary Figure 5. AAV2 IgM/IgG levels over time in a) individual cases and b) controls 21

Supplementary Figure 6. Quantification of immune cells in liver cases and adult controls 22

Supplementary Figure 7. Quantification of positively stained cells using CODEX-technology 23

Supplementary clinical information

1.1. Approach to clinical investigation and diagnosis

In response to an outbreak of acute hepatitis in children, Public Health Scotland (PHS) convened a National Incident Management Team (IMT), alongside the UK Health Security Agency (UKHSA) and academic partners (International Severe Acute Respiratory Infection Consortium Clinical Characterisation Collaboration (ISARIC4C) Investigators) to coordinate further investigations and the public health response. The working PHS case definition was “a serum transaminase of >500IU/L (AST or ALT) without any known viral or alternative cause, in children aged 10 years or under presenting after 1st January 2022”. As of 30th September 2022, 44 children aged 10 years and under with acute unexplained non-A to E hepatitis were confirmed in Scotland, one of whom required liver transplantation.[3] The epidemiological investigation of this case cluster was led by Public Health Scotland and included detailed trawling questionnaires which determined no common exposures.

A list of recommended clinical investigations, including extensive virology testing, autoantibody screening, toxicology and other causes of hepatitis was determined by a multi-disciplinary working group led by Public Health Scotland. Since some cases were retrospectively identified, and due to technical difficulties in sampling from young children, not all investigations were completed in all patients.

Clinical referral pathways and guidelines were established across Scotland. A peer review forum (comprised of paediatric gastroenterologists, general paediatricians with a gastroenterology special interest and a paediatric infectious diseases specialist) was established to discuss patients. A representative referral pathway with investigation list can be found here: https://www.clinicalguidelines.scot.nhs.uk/nhsggc-guidelines/nhsggc-guidelines/infectious-disease/west-of-scotland-paediatric-hepatitis-pathway/. In complex or borderline cases, the peer review forum was used to agree whether an alternative cause for the clinical presentation was likely.

1.2. Comorbidities and prior medication

9/32 (28%) of children had at least one known co-morbidity. Comorbidities comprised two children with chromosomal disorders with complex co-morbidity, one child with each of the following: ornithine transcarbamylase deficiency; attention deficit hyperactivity disorder (ADHD; previously on methylphenidate and melatonin, not currently on medication); recent but resolved haemolytic uraemic syndrome; alopecia and previous autoimmune IgA nephropathy; asthma and eczema; multiple food allergy. One child was two years post-liver transplant for a non-autoimmune familial liver disease, liver function was stable prior to the episode of hepatitis and resolved without treatment.

Only children with known co-morbidities were receiving regular medications prior to admission. Of these, four children were receiving regular medications associated with hepatic adverse effects. These respectively comprised; esomeprazole and spironolactone; tacrolimus and mycophenolate mofetil; lanreotide; montelukast. Hepatitis was not considered as likely secondary to drug effects in these cases and resolved despite these medications being continued. Several children received paracetamol prior to admission, none in excess of the therapeutic dose and none with raised paracetamol level.

1.3. Autoantibody testing

The ANA screens were performed on HEp-2 cells, an indirect immunofluorescence assay (IIF) with a screening dilution of 1:80. If the ANA screen was positive then an ANA titration was performed. Results for each patient were reported as either negative or positive (a positive result includes titre and pattern). Liver autoantibody screen was performed on rodent liver/kidney/stomach tissue, by IIF. Most laboratories across Scotland use a screening dilution of 1:40. One immunology laboratory which tested samples in this cohort screened paediatric samples at 1:10. Results for each patient were reported as either negative or positive (a positive result included a pattern and, where appropriate, titre).

1.4. Ethical approvals

Ethical approval for recruitment of cases under the ISARIC WHO Clinical Characterisation Protocol UK (CCP-UK) [ISRCTN 66726260] was given by the South Central–Oxford C Research Ethics Committee (REC) in England (13/SC/0149), the Scotland A REC (20/SS/0028), and the WHO Ethics Review Committee (RPC571 and RPC572).

The DIAMONDS study (https://www.diamonds2020.eu) was approved by the London – Dulwich REC (20/HRA/1714). Patients were recruited with the written informed consent of parents or guardians.

Contemporaneous Scottish surplus plasma and liver biopsy control samples from the Diagnostic Pathology/Blood Sciences archive were obtained with NHS GG&C Biorepository approval (application #717; REC 22/WS/0020). These samples were used without consent following HTA legislation on consent exemption.

Genetic (HLA) control data was obtained using the UK Biobank Resource (project 788; REC 21/NW/0157).

Adult liver biopsy samples used for IHC and CODEX were obtained commercially.

Residual sample testing Residual plasma or serum samples were tested in cases by NGS and by PCR for the presence of AAV2. Group 1 healthy controls were all serum samples, Group 2 included 8 plasma and 5 sera, Group 3 consisted of 27 sera and 9 plasma and Group 4 were all plasma. We tested Ct values and GAPDH in a small number of paired serum/plasma samples and found these to be similar to ensure consistency by sample type (data not shown).

1. Supplementary Tables

Supplementary Table 1. Clinical virology results of the 32 non-A-E hepatitis cases in Scottish children

Patient		1	2	3	4	5	6	7	8
**HAdV PCR**	**Plasma**	Negative	Negative	Negative	Negative	Negative	Negative	Negative	Negative
	**Throat/nose**	NT	Negative	Positive (Ct 38)	Negative	Negative	Negative	Negative	Positive (Ct 30)
	**Stool**	Negative	Negative	Negative	Negative	Positive (Ct 31)	Positive (Ct 34)	Positive (Ct 29)	Positive (Ct 31)
**Hepatitis A**	**IgM**	Negative	Negative	Negative	Negative	Negative	Negative	Negative	Negative
	**PCR**	Negative	Negative	Negative	Negative	Negative	NT	NT	NT
**Hepatitis B**	**sAg**	Negative	Negative	Negative	Negative	Negative	Negative	Negative	Negative
**Hepatitis C**	**PCR**	Negative	NT (IgG negative)	Negative	Negative	Negative	Negative	NT	NT
**Hepatitis E**	**IgM**	Negative	Negative	Negative	Negative	Negative	Negative	Negative	Negative
	**PCR**	Negative	Negative	Negative	Negative	Negative	NT	NT	NT
**Parvovirus B19**	**IgM**	Negative	NT	Negative	Negative	Negative	Negative	Negative	Negative
	**PCR**	NT	Negative	NT	NT	NT	NT	NT	NT
**CMV**	**IgM**	Negative	Negative	Negative	Negative	Negative	Negative	Negative	Negative
**EBV**	**IgM**	Negative	Negative	Negative	Negative	Negative	Negative	Negative	Negative
**Enterovirus**	**Plasma PCR**	Negative	Negative	Negative	Negative	Negative	NT	NT	Negative
**HSV**	**Plasma PCR**	Negative	Negative	Negative	Negative	Negative	Negative	Negative	Negative
**HIV**	**Plasma/ serum Ag/Ab**	Negative	Negative	NT	Negative	Negative	Negative	Negative	Negative
**HHV6/7**	**Blood PCR**	Negative	Negative	Negative	Negative	Negative	Negative	Negative	Negative
**Respiratory viral**	**Throat/nose PCR**	NT	Negative	HCoV-NL63 (Ct 34)	Negative	Paraflu 3	Paraflu 2 (Ct 34) Rhino/EV (Ct 28)	Negative	Rhino/EV (Ct 36)
**SARS-CoV-2**	**Throat/nose PCR**	Negative	Negative	Negative	Negative	Positive	Positive	Negative	Negative
	**Plasma**<br>**S Ab**<br>**N Ab**	NT<br>NT	Positive<br>Positive	NT<br>NT	Positive<br>Positive	Positive<br>Positive	NT<br>NT	Negative<br>Negative	Negative<br>Negative
**Liver**	**CMV, EBV, HSV, HHV6/7 PCR**	HHV6 (Ct 33)		Negative	HHV6 (Ct 36)				
**Other sample**								Stool Norovirus (Ct 27)	

Patient		9	10	12	13	14	15	16	17
**HAdV PCR**	**Plasma**	Negative	Negative	Negative	NT	Positive (Ct 37)	Positive (Ct 39.9)	Positive* (Ct 35)	Negative
	**Throat/nose**	NT	Negative	Negative	Positive (Ct N/A)	Negative	Negative	Negative	Positive (Ct 37)
	**Stool**	NT	NT	Negative	Positive (Ct 29)	Positive (Ct 35)	Negative	Negative	Positive (Ct 34)
**Hepatitis A**	**IgM**	Negative	Negative	Negative	Negative	Negative	Negative	Negative	Negative
	**PCR**	NT	Negative	NT	NT	NT	NT	NT	NT
**Hepatitis B**	**sAg**	Negative	Negative	Negative	Negative	Negative	Negative	Negative	Negative
**Hepatitis C**	**PCR**	Negative	Negative	NT (IgG Negative)	NT (IgG Negative)	NT (IgG Negative)	NT (IgG Negative)	NT (IgG Negative)	Negative
**Hepatitis E**	**IgM**	Negative	Negative	Negative	Negative	Negative	Negative	Negative	Negative
	**PCR**	NT	NT	NT	NT	NT	NT	NT	NT
**Parvovirus B19**	**IgM**	NT	Negative	Negative	NT	Negative	Negative	NT	NT
	**PCR**	NT	NT	NT	NT	NT	NT	NT	NT
**CMV**	**IgM**	Negative	Negative	Negative	Negative	Negative	Negative	Negative	Negative
**EBV**	**IgM**	Negative	Negative	Negative	Negative	Negative	Negative	Negative	Equivocal*
**Enterovirus**	**Plasma PCR**	NT	Negative	NT	NT	Negative	Negative	Negative	Negative
**HSV PCR**	**Plasma PCR**	Negative	Negative	NT	NT	Negative	Negative	Negative	Negative
**HIV Ag/Ab**	**Plasma/ serum Ag/Ab**	NT	NT	NT	NT	NT	NT	Negative	Negative
**HHV6/7**	**Plasma PCR**	Negative	HHV6 (Ct 37)	NT	NT	Negative	Negative	NT	Negative
**Respiratory viral**	**Throat/ nose PCR**	NT	Negative	Negative	Negative	Negative	Coronavirus HKU1; Rhinovirus	Negative	Negative
**SARS-CoV-2**	**Throat/ nose PCR**	Negative	Negative	Negative	Negative	Positive (Ct 29)	Negative	Negative	Negative
	**Blood**<br>**S Ab**<br>**N Ab**	 NT<br>NT	 Positive<br>Negative	 Positive<br>Positive	 Positive<br>Negative	 NT<br>Positive	 Positive<br>Positive	 Positive<br>Negative	 Positive<br>Positive
**Liver**	**CMV, EBV, HSV, HHV6/7 PCR**	Negative							
**Other sample**				Stool Enterovirus (Ct 34)	Stool Norovirus	Stool Sapovirus			

*EBVVCA IgG positive & EBNA IgG positive suggesting past infection

Patient		18	19	20	22	23	24	26	27
**HAdV PCR**	**Plasma**	Positive (Ct 35)	Negative	Negative	Negative	Positive (Ct 30)	Negative	Negative	Negative
	**Throat/nose**	Negative	Negative	Negative	Negative	Negative	Positive (Ct 31)	Negative	NT
	**Stool**	Negative	Negative	Negative	Negative	NT	Positive (Ct 31)	Negative	NT
**Hepatitis A**	**IgM**	Negative	Negative	Negative	Negative	Negative	Negative	Negative	Negative
	**PCR**	NT	NT	NT	NT	NT	NT	NT	NT
**Hepatitis B**	**sAg**	Negative	Negative	Negative	Negative	Negative	Negative	Negative	Negative
**Hepatitis C**	**PCR**	NT (IgG Negative)	NT (IgG Negative)	NT (IgG Negative)	NT (IgG Negative)	NT (IgG Negative)	NT (IgG Negative)	NT (IgG negative)	Negative
**Hepatitis E**	**IgM**	Negative	Negative	NT	Negative	Negative	Negative	Negative	Negative
	**PCR**	NT	NT	NT	NT	NT	NT	NT	NT
**Parvovirus B19**	**IgM**	NT	NT	NT	NT	NT	NT	NT	NT
	**PCR**	NT	NT	Negative			NT	NT	NT
**CMV**	**IgM**	Negative	Negative	Negative	Negative	Negative	NT	Negative	Negative
**EBV**	**IgM**	Negative	Negative	Negative	NT<br>PCR negative	Negative	NT	Negative	Negative
**Enterovirus PCR**	**Plasma**	NT	NT	NT	NT	NT	NT	NT	Negative
**HSV PCR**	**Plasma**	Negative	Negative	Negative	NT	NT	NT	HSV1 (Ct 25)	NT
**HIV Ag/Ab**	**Plasma /serum**	NT	NT	Negative	NT	NT	NT	NT	Negative
**HHV6/7**	**Plasma PCR**	Negative	NT	Negative	Negative	Negative	Negative	HHV7 (Ct 35)	Negative
**Respiratory viral**	**Throat/ nose PCR**	Negative	Negative	Rhinovirus	Negative	Negative	RSV (Ct 37)	Rhinovirus/ EV (Ct 29)	Negative
**SARS-CoV-2**	**PCR Throat/ nose**	Negative	Negative	Negative	Negative	Negative	Negative	Negative	Negative
	**S Ab**<br>**N Ab**	Positive<br>Positive	Positive<br>Positive	NT<br>NT	Positive<br>Positive	Positive<br>NT	NT<br>NT	NT<br>NT	NT<br>NT
**Liver**	**CMV, EBV, HSV, HHV6/7 PCR**			Negative (EBV PCR NT)					
**Other sample**			Stool – Enterovirus PCR positive (Ct 22)		Stool Parechovirus (Ct 31)		Stool Norovirus (Ct 21)		

Patient		28	29	30	32	34	35	36	37
**HAdV PCR**	**Plasma**	Negative	NT	Negative	Negative	Negative	Negative	Negative	Negative
	**Throat/nose**	NT	NT	NT	Negative	NT	NT	Negative	Negative
	**Stool**	NT	NT	Negative	Negative	Negative	NT	Negative	NT
**Hepatitis A**	**IgM**	Negative	Negative	Negative	Negative	Negative	Negative	Negative	Negative
	**PCR**	NT	NT	Negative	NT	NT	NT	NT	NT
**Hepatitis B**	**sAg**	Negative	Negative	Negative	Negative	Negative	Negative	Negative	Negative
**Hepatitis C**	**PCR**	Negative	NT	NT (IgG negative)	Negative	NT (IgG Negative)		NT (IgG negative)	NT (IgG negative)
**Hepatitis E**	**IgM**	Negative	NT	Negative	Negative	Negative	Negative	NT	Negative
	**PCR**	NT	NT	Negative	NT	NT	Negative	NT	NT
**Parvovirus B19**	**IgM**	NT	NT	NT	NT	NT	NT	Negative	Negative
	**PCR**	NT	NT	NT	NT	NT	Negative	NT	NT
**CMV**	**IgM**	Negative	Negative	NT	Negative	Negative	NT	Negative	Negative
**EBV**	**IgM**	Negative	Negative	NT	Negative	Low positive*	Positive†	Negative	Negative
**Enterovirus PCR**	**Plasma PCR**	Negative	NT	NT	NT	NT	Equivocal	NT	NT
**HSV PCR**	**Plasma PCR**	Negative	NT	Negative	Negative	NT	Negative	Negative	Negative
**HIV Ag/Ab**	**Plasma/ serum Ag/Ab**	NT	Negative	Negative	NT		Negative	NT	NT
**HHV6/7**	**Plasma PCR**	Negative	NT	Negative	Negative	Negative	Negative	Negative	HHV6 negative, HHV7 positive
**Respiratory viral PCR***	**Throat/nose PCR**	Flu A/B negative	Flu A/B/RSV negative	Flu A/B/RSV negative	Negative	NT	NT	Negative	All negative
**SARS-CoV-2 PCR**	**Throat/nose PCR**	Negative	Negative	Negative	Negative	NT	Negative	Negative	Negative
	**Plasma**<br>**S Ab**<br>**N Ab**	 NT<br>NT	 NT<br>NT	 NT<br>NT	 NT<br>NT	 NT<br>NT	 NT<br>NT	 NT<br>NT	 NT<br>NT
**Liver**	**CMV, EBV, HSV, HHV6/7 PCR**								
**Other sample**								non O157 E. coli in stool	

Abbreviations – PCR, polymerase chain reaction; CMV, cytomegalovirus; EBV, Epstein-Barr Virus; EV, Enterovirus; HSV, Herpes Simplex virus; HIV, Human Immunodeficiency Virus; HHV, Human herpesvirus; PCR, polymerase chain reaction; NT, not tested. *EBVVCA IgG & EBNA IgG negative. † No EBV IgG results available.

Supplementary Table 2. Non-virological clinical results of the 32 non-A-E hepatitis cases in Scottish children

Patient	1	2	3	4	5	6	7	8
Sex	F	M	M	F	F	M	F	F
Age	3y 6m	5y 6m	4y 7m	3y 7m	4y 2m	3y 11m	1y 9m	3y 4m
Peak bilirubin (umol/L)	221	132	387	363	76	27	18	142
Peak ALT (U/L)	2743	2516	3387	3874	640	2080	1603	2907
Peak AST (U/L)	3570	2289	5401	6160	1404	2926	1389	2881
Peak ALP (U/L)	381	243	492	539	342	288	212	326
Peak GGT (U/L)	124	112	126	126	453	141	97	339
Peak INR	1.4	1.0	1.9	2.9	1.4	1.2	1.0	1.0
Peak CRP (mg/L)	10	1	4	26	7	5	1	4
Caeruloplasmin (g/L)	0.38	0.39	0.4	0.52	0.29	0.36	NT	0.43
IgG (g/L)	12.6	12.9	9.9	13.7	21	11.5	10.1	14.4
TTG Ab (U/ml)	0.3	0.5	0.8	0.5	1.6	0.5	<0.1	0.4
Mitochondrial Ab	Negative	Negative	Negative	Negative	Negative	Negative	Negative	Negative
Smooth muscle Ab	Negative	Negative	Negative	Negative	Negative	Negative	Negative	Negative
LKM Ab	Negative	Negative	Negative	Negative	Negative	Negative	Negative	Negative
ANA	Negative	Negative	Negative	Negative	Negative	Negative	Negative	1:80 homogenous

Patient	9	10	12	13	14	15	16	17
Sex	M	M	M	F	M	F	F	M
Age	10y 7m	2y 6m	3y 5m	11m	3y 8m	2y 2m	5y 1m	6y 4m
Peak bilirubin (umol/L)	39	49	80	3	214	58	176	265
Peak ALT (U/L)	2343	2122	1308	525	1406	1266	3069	3689
Peak AST (U/L)	2211	1908	1237	NT	NT	737	3858	4047
Peak ALP (U/L)	203	376	318	190	505	717	325	470
Peak GGT (U/L)	171		135	163	88	440	123	65
Peak INR	1.1	1.1	1.1	1.1	1.6	1.1	1.3	1.4
Peak CRP (mg/L)	4	1	1	4	5	3		<6
Caeruloplasmin (g/L)	0.34	0.29	NT	NT	NT	0.36	0.39	0.37
IgG (g/L)	16.4	8.7	1.5	2.62	11.62	6.18	12.3	10.6
TTG Ab (U/ml)	0.5	<0.1	Negative	NT	NT	0.39	0.6	0.7
Mitochondrial Ab	Negative	Negative	Negative	Negative	Negative	Negative	Negative	Negative
Smooth muscle Ab	Negative	Negative	Negative	Negative	Negative	Negative	Negative	Negative
LKM Ab	Negative	Negative	Negative	Negative	Negative	Negative	Negative	Negative
ANA	Negative	Negative	Negative	Negative	Negative	Negative	Negative	1:80 speckled

Patient	18	19	20	22	23	24	26	27
Sex	F	F	F	F	F	M	F	M
Age	3y 1m	6y 3m	5y 11m	9y 6m	4y 7m	5y 6m	6y 7m	2y 1m
Peak bilirubin (umol/L)	95	55	55	11	93	6	18	5
Peak ALT (U/L)	1153	594	1766	674	1747	674	719	633
Peak AST (U/L)	1121	572	2188	521	1361	579	436	508
Peak ALP (U/L)	532	684	440	217	561	523	144	273
Peak GGT (U/L)	93	359	101	67	111	170	18	24
Peak INR	1.2	1.5	1.3	1.0	1.0	1.0	1.0	NT
Peak CRP (mg/L)	3	16	6	21	<5	2	11	8
Caeruloplasmin (g/L)	0.41	0.22	NT	0.35	0.42	NT	0.24	0.33
IgG (g/L)	9.9	4.3	18.4	14.2	15.9	NT	4.5	9.2
TTG Ab (U/ml)	1.7	0.51	NT	0.5	0.4	NT	0.2	0.3
Mitochondrial Ab	Negative	Negative	Negative	Negative	Negative	Negative	Negative	Negative
Smooth muscle Ab	Negative	Negative	1:40	Negative	Negative	Negative	Negative	Negative
LKM Ab	Negative	Negative	Negative	Negative	Negative	Negative	Negative	Negative
ANA	Negative	Negative	Negative	Negative	Negative	Negative	Negative	Negative

Patient	28	29	30	32	34	35	36	37
Sex	F	F	F	F	F	F	F	M
Age	5y 2m	4y 6m	5y 7m	2y 7m	2y 8m	2y 1m	2y 8m	7y 4m
Peak bilirubin (umol/L)	154	222	64	129	108	238	84	6
Peak ALT (U/L)	4101	2822	1442	2031	2282	5417	333	667
Peak AST (U/L)	3568	3914	1321	2527	2482	6908	561	424
Peak ALP (U/L)	280	528	337	401	271	572	1007	212
Peak GGT (U/L)	161	74	53	134	125	119	720	33
Peak INR	1.3	1.4	1.1	1.2	1.1	2.5	1.3	1.1
Peak PT (sec)	14.6	14	12.6	17.5	14.2	NT	15.3	13.1
Peak CRP (mg/L)	<6	11	<6	41	6	11	117	4
Caeruloplasmin (g/L)	0.34	0.35	0.39	0.33	NT	0.35	NT	0.24
IgG (g/L)	14.9	12.8	15.1	11.8	14.2	20.7	10.6	10.9
TTG Ab (U/ml)	NT	0.6	0.3	0.5	0.3	Negative	NT	0.6
Mitochondrial Ab	Negative	Negative	Negative	Negative	Negative	Negative	Negative	Negative
Smooth muscle Ab	Negative	Negative	Negative	Negative	Negative	Negative	1:40	1:40
LKM Ab	Negative	Negative	Negative	Negative	Negative	Negative	Negative	Negative
ANA	1:80 speckled	Negative	1:80 homogenous	Negative	Negative	Negative	Negative	Negative

Abbreviations: Ab, antibody; ALT, alanine transaminase; AST, Aspartate transaminase; ALP, alkaline phosphatase; GGT, gamma-glutamyl transferase; INR, international normalised ratio; PT, prothrombin time; CRP, C-reactive protein; IgG, immunoglobulin G; TTG, Tissue Transglutaminase; LKM, liver-kidney microsomal; ANA, anti-nuclear antibody; NT, not tested.

Supplementary Table 3. Read counts per million of AAV2 and HAdV in cases following target enrichment sequencing

Patient number		1	2	3	4	5	6	7	8	9
**Serum**										
Day of infection		51	33	21	26	9	54	80	15	15
	DNA	10327.10	774.15	10498.28	24967.81	152.06	4477.52	401.41	1047.57	23572.34
	RNA	133.25		12119.78	1016.67		380.98		805.89	3717.11
	DNA	0.00	0.00	1.45	1.66	0.00	0.00	0.60	0.00	0.00
	RNA	0.00	0.00	677.57	0.00	0.00	0.00	0.00	0.00	0.00
**Liver**										
Day of infection		54		28	30					7
	DNA	31360.12		10372.68	39653.48					637.16
	RNA	3089.09								
	DNA	0.00		419.33	678.08					1349.78
	RNA	0.00		0.00	0.00					0.00
**Faeces**										
Day of infection		52		22	23	10	59	80	15	
	DNA	0.00		0.00	0.00	3156.64	0.00	0.00	0.00	
	RNA	0.00		0.00	0.00	544.03	0.00	0.00	0.00	
	DNA	0.00		0.72	0.00	10512.00	0.00	23.36	3490.31	
	RNA	0.00		0.00	0.00	0.00	0.00	0.00	0.00	
**Rectal**										
Day of infection								80		
	DNA							108.68		
	RNA							0.00		
	DNA							2.93		
	RNA							0.00		
**Throat**										
Day of infection				21	31	13	54	80	15	
	DNA			0.00	670.26	0.00	0.00	0.00	0.00	
	RNA			0.00	0.00	0.00	0.00	0.00	0.00	
	DNA			132.96	0.00	0.00	0.00	0.00	14453.17	
	RNA			0.00	0.00	0.00	0.00	0.00	0.00	

Key

0 >100

0 >10

Supplementary Table 4. Read counts per million of HHV in case and control sera following target enrichment sequencing

Patient ID			1	2	3	4	5	6	7	8	9	10	11	12	13
Cases		DNA						0.00	0.00	0.00	0.00				
	HSV1	RNA						0.00	0.00	0.00	0.00				
		DNA						0.00	0.00	0.00	0.00				
	HSV2	RNA						0.00	0.00	0.00	0.00				
		DNA						0.00	2.14	0.00	0.00				
	VZV	RNA						0.00	0.00	0.00	0.00				
		DNA						0.53	0.00	0.00	0.00				
	EBV	RNA						0.00	0.00	0.00	0.00				
		DNA						0.00	0.00	9.85	0.00				
	CMV	RNA						0.00	0.00	0.00	0.21				
		DNA						0.00	0.00	0.00	0.00				
	HHV6A	RNA						0.00	0.00	0.00	0.00				
		DNA						3.39	3.89	0.00	0.41				
	HHV6B	RNA						0.00	0.00	0.00	0.00				
		DNA						0.00	0.00	0.00	0.00				
	HHV7	RNA						0.00	0.00	0.31	0.00				
		DNA						0.00	0.00	0.00	0.00				
	HHV8	RNA						0.00	0.00	0.00	0.00				
Adenovirus positive controls		DNA	0.00	3.35	0.00	0.00	0.00	0.00	0.00	0.00	0.00	0.00	0.00	0.00	
	HSV1	RNA	0.00	11.38	0.00	0.00	0.00	0.00	0.00	0.00	0.00	0.00	0.00	0.00	
		DNA	0.00	10.37	0.00	0.00	0.00	0.00	0.00	0.00	0.00	0.00	0.00	0.00	
	HSV2	RNA	1.00	22.92	0.00	0.00	0.00	0.00	0.00	0.00	0.00	0.00	0.00	0.00	
		DNA	0.00	2.68	0.00	0.00	0.00	0.00	0.00	0.00	0.00	0.00	0.00	0.00	
	VZV	RNA	0.00	4.02	0.00	0.00	0.00	0.00	0.00	0.00	0.00	0.00	0.00	0.00	
		DNA	0.00	167.63	0.00	0.00	0.00	0.00	0.00	0.00	1.64	0.00	272.40	0.00	
	EBV	RNA	0.00	9.03	0.00	0.00	0.00	0.00	0.00	0.00	0.00	0.00	1.38	17.58	
		DNA	0.00	3634.02	56.82	123.87	0.00	8.26	0.00	0.00	25.24	0.00	0.00	0.00	
	CMV	RNA	0.00	3185.33	0.00	0.00	0.00	0.00	0.00	0.00	0.00	0.00	0.00	0.00	
		DNA	0.00	1.67	0.44	0.00	0.00	0.00	0.00	0.00	0.00	0.00	0.00	0.00	
	HHV6A	RNA	0.00	12.38	0.00	0.00	0.00	0.00	0.00	0.00	0.00	0.00	0.00	0.00	
		DNA	0.00	5062.23	0.00	0.00	0.00	0.00	0.00	0.00	0.00	1050.19	0.00	0.18	
	HHV6B	RNA	0.00	5819.09	0.00	0.00	0.00	0.00	0.00	0.00	0.00	206.17	0.00	0.00	
		DNA	0.00	0.00	0.00	0.00	0.00	0.00	0.00	0.00	0.00	0.00	0.00	0.00	
	HHV7	RNA	2.00	10.54	0.00	0.00	0.00	0.00	0.00	0.00	0.00	0.00	0.00	0.00	
		DNA	0.00	2.01	75.91	0.00	0.00	0.00	0.00	0.00	0.00	0.00	0.00	0.00	
	HHV8	RNA	0.00	1.34	0.00	0.00	0.00	0.00	0.00	0.00	0.00	0.00	0.00	0.00	
Healthy controls		DNA	0.00	0.00	0.00	0.00	0.00	0.00	0.00	0.00	0.00	0.00	0.00	0.00	0.00
	HSV1	RNA	0.00	0.00	0.00	0.00	0.00	0.00	0.00	0.00	0.00	0.00	0.00	0.00	0.00
		DNA	0.00	0.00	0.00	0.00	0.00	0.00	0.00	0.00	0.00	0.00	0.00	0.00	0.00
	HSV2	RNA	0.00	0.00	0.00	0.00	0.00	0.00	0.00	0.00	0.00	0.00	0.00	0.00	0.00
		DNA	0.00	0.00	0.00	0.00	0.00	0.00	0.00	0.00	0.00	0.00	0.00	0.00	0.00
	VZV	RNA	0.00	0.00	0.00	0.00	0.00	0.00	0.00	0.00	0.00	0.00	0.00	0.00	0.00
		DNA	0.00	0.00	0.00	0.00	0.00	0.00	0.00	0.00	1.23	13.01	114.38	0.00	0.00
	EBV	RNA	1.00	0.00	0.00	0.00	0.00	0.00	0.00	0.10	0.00	0.00	0.00	0.18	0.00
		DNA	0.00	17.06	0.00	0.00	0.00	0.00	0.00	0.00	0.00	0.00	0.00	0.00	0.00
	CMV	RNA	0.00	0.00	0.00	0.00	0.00	0.00	0.00	0.00	0.00	0.00	0.00	0.00	0.00
		DNA	0.00	0.00	0.00	0.00	0.00	0.00	0.00	0.00	0.00	0.00	0.00	0.00	0.00
	HHV6A	RNA	0.00	0.00	0.00	0.08	0.00	0.00	0.10	0.00	0.00	0.00	0.00	5.10	0.00
		DNA	0.00	0.00	0.00	0.00	0.00	0.00	0.00	0.00	0.00	0.00	0.00	0.00	0.00
	HHV6B	RNA	0.00	0.00	0.00	0.00	0.00	0.00	0.00	0.00	0.00	0.00	0.00	0.00	0.00
		DNA	0.00	0.00	0.00	0.00	0.00	0.00	0.00	0.00	0.00	0.00	0.00	0.00	0.00
	HHV7	RNA	0.00	0.00	0.00	0.00	0.00	0.00	0.00	0.10	0.00	0.00	0.00	0.00	0.00
		DNA	0.00	0.00	0.00	0.00	0.00	0.00	0.00	0.00	0.00	0.00	0.00	0.00	0.00
	HHV8	RNA	0.00	0.00	0.00	0.00	0.00	0.00	0.00	0.00	0.00	0.00	0.00	0.00	0.00

Reads per million
0 >10

Supplementary Table 5. Read counts per million of AAV2 and HAdV in case and control sera following target enrichment sequencing

Patient ID		1	2	3	4	5	6	7	8	9	10	11	12	13
Day of infection		51	33	21	26	9	54	80	15	15				
**Adeno-associated virus 2**														
Case	DNA	10327.10	774.15	10498.28	24967.81	152.06	4477.52	401.41	1047.57	23572.34				
	RNA	133.25	0.00	12119.78	1016.67	0.00	380.98	35.21	805.89	3717.11				
Adenovirus positive control	DNA	0.00	0.00	0.00	0.00	0.00	0.00	0.00	0.00	0.00	0.00	0.00	0.00	
	RNA	0.00	0.00	0.00	0.00	0.00	0.00	0.00	0.00	0.00	0.00	0.00	0.00	
Healthy control	DNA	0.00	0.00	0.00	0.00	0.00	0.00	0.00	0.00	0.00	0.00	0.00	0.00	0.00
	RNA	0.00	0.00	0.00	0.00	0.00	0.00	0.00	0.00	0.00	0.00	0.00	0.00	0.00
**Adenovirus C or F**														
Case	DNA	0.00	0.00	1.45	1.66	0.00	0.00	0.60	0.00	0.00				
	RNA	0.00	0.00	677.57	0.00	0.00	0.00	0.00	0.00	0.00				
Adenovirus positive control	DNA	0.00	1.63	0.00	29950.78	0.00	16362.76	2860.62	0.00	347.34	450.33	0.00	0.00	
	RNA	0.00	0.00	0.00	13.74	0.00	0.00	0.00	0.00	0.00	2.36	0.00	0.00	
Healthy control	DNA	0.00	0.00	0.00	0.00	0.00	0.00	0.00	0.00	0.00	0.00	0.00	0.00	0.00
	RNA	0.00	0.00	0.00	0.00	0.00	0.00	0.00	0.00	0.00	0.00	0.00	0.00	0.00

Supplementary Table 6. Antibodies used for immunohistochemistry

Antigen	Dilution	Clone	Product code, company	Antigen retrieval	Detection system
MHCII	1:200	none	M0746, Dako/Agilent	Pressure cooking; citrate pH6	Envision Dako Agilent
C4d complement	1:100	none	Quidel A213 Antihuman C4d	ER2 (20)	Leica BOND polymer DS9800 and BOND DAB enhancer
CD3	1:100	LN10	Leica NCL-L-CD3-565	ER2 (20)	Leica BOND polymer DS9800 and BOND DAB enhancer
CD4	1:200	1F6	Leica NCL-L-CD4-368	ER2 (20)	Leica BOND polymer DS9800 and BOND DAB enhancer
CD8	1:50	4B11	Leica NCL-CD8-4B11	ER2 (20)	Leica BOND polymer DS9800 and BOND DAB enhancer
CD20	1:200	L26	Novocastra NCL-L-CD20-L26	ER1 (20)	Leica BOND polymer DS9800 and BOND DAB enhancer

Supplementary Table 7. Panel for Codex analysis

Target	Ref #	Supplier	Reporter/Barcode	Fluorophore	Concentration
CD20	4450018	Akoya Biosciences	Bx007	AF750	1/200
CD44	4450041	Akoya Biosciences	Bx005	Atto 550	1/100
CD3	4450030	Akoya Biosciences	Bx045	Cy5	1/200
PanCK	4450020	Akoya Biosciences	Bx019	AF750	1/200
CD31	4450017	Akoya Biosciences	Bx001	AF750	1/100
Mx1	M143	Custom made- University Medical Centre Freiburg	Bx022	AF 750	1/50
CD8	4250012	Akoya Biosciences	BX026	Atto 550	1/200
CD68	4350019	Akoya Biosciences	Bx015	Cy5	1/200
CD107a	4350001	Akoya Biosciences	Rx006	Cy5	1/200
CD4	4350018	Akoya Biosciences	Bx003	cy5	1/200

2. Supplementary Figures

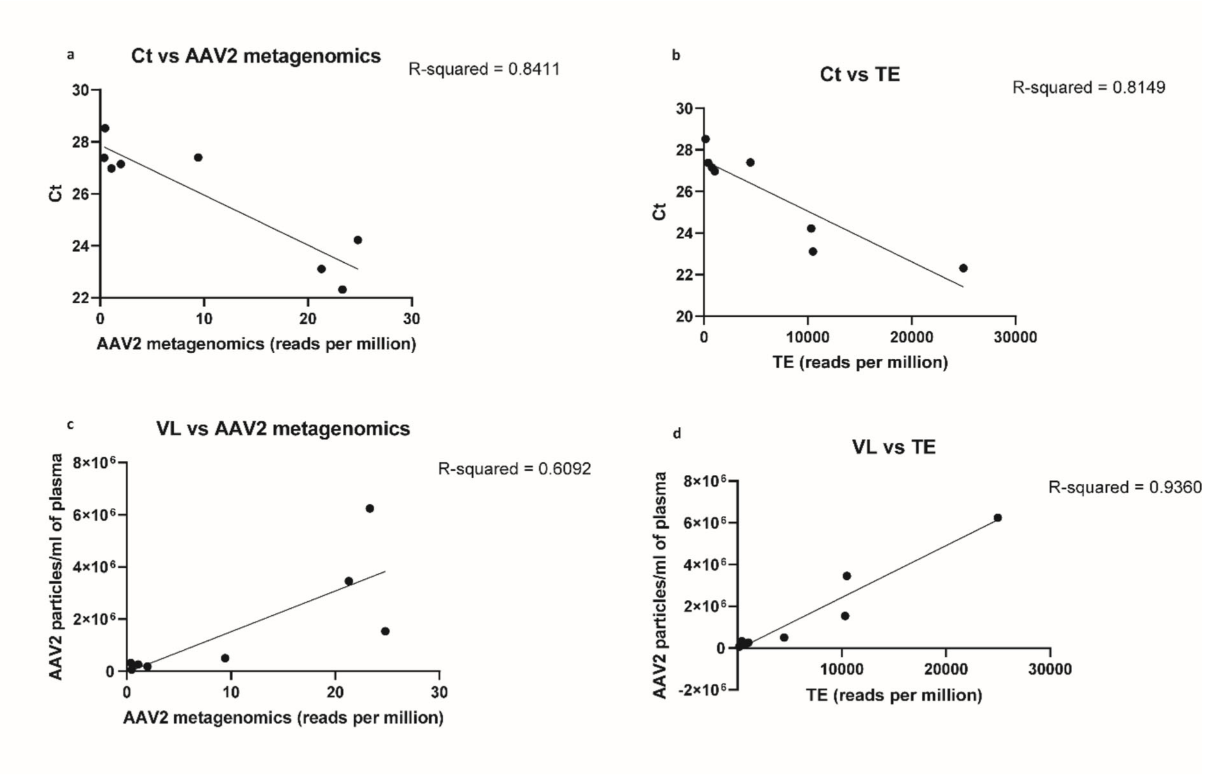

Supplementary Figure 1. AAV2 NGS read counts plotted against RT-PCR cycle Threshold (Ct) and estimated viral loads a) Comparison of median RT-PCR Ct values versus median AAV2 metagenomic read counts; **b**) RT-PCR Ct values versus target enrichment (TE) read counts; **c)** Metagenomic **AAV2** NGS read counts versus median RT-PCR estimated viral load (VL) and; **d**, Median target enrichment (TE) read counts versus median viral load (VL) . The correlation co-efficient squared (R^2) is indicated in the top right of each panel.

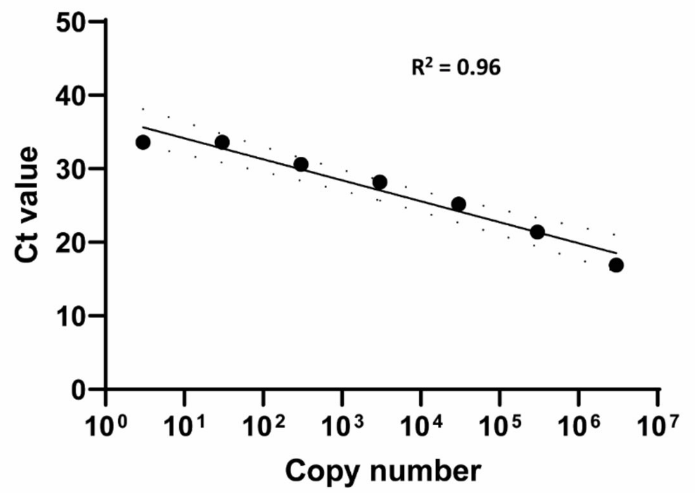

Supplementary Figure 2. Cycle threshold (Ct) values and estimated copy number for AAV2 real-time PCR. Estimated copy numbers are shown, calculated using 7 serial dilutions of a plasmid containing the 62bp ITR product to generate a standard curve which was then used to calculate the copy number of AAV2 in triplicate samples (median values are displayed as large dots). The correlation co-efficient squared (R^2) was 0.96.

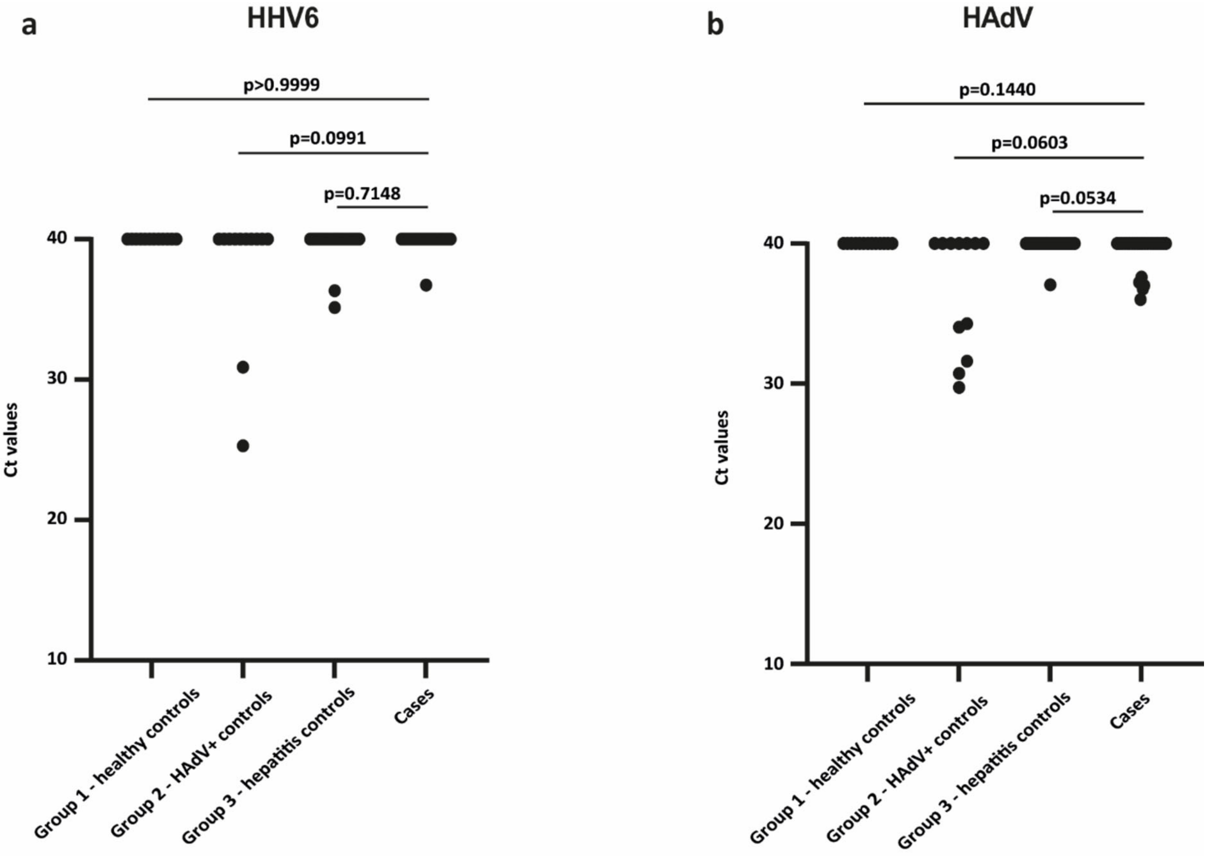

Supplementary Figure 3. Cycle threshold (Ct) values for cases and controls for HHV6 and HAdV PCR. Ct values are shown for cases and controls tested for **a)** HHV6 **and b)** HAdV. Statistical significance was estimated using a Mann-Whitney test (two-sided). PCR testing was carried out on one occasion in 28 cases from whom residual samples were available and all 74 controls (Groups 1,2,3,4) using validated assays in the NHS GG&C West of Scotland Specialist Virology Centre.

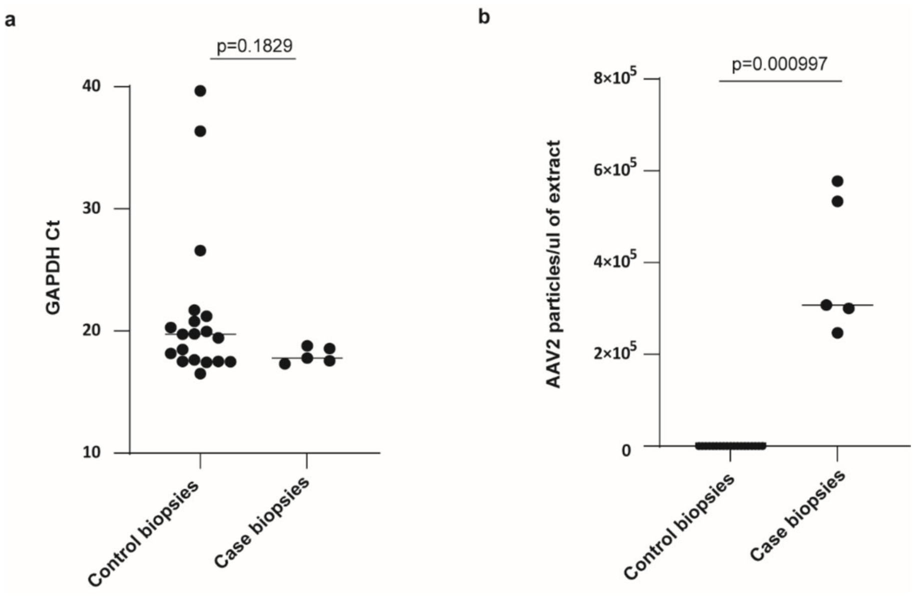

Supplementary Figure 4. GAPDH values for liver biopsy cases and controls. GAPDH Ct values are shown for liver biopsy cases (n=5) and controls (n=19) in panel **a,** and AAV2 PCR results after removal of outlier GAPDH control samples in panel **b.** Cases were tested in triplicate. Controls were tested in duplicate (n=17) or triplicate (n=2) depending on sample and reagent availability. Median values are shown. Statistical significance was estimated using a Mann-Whitney test (two-sided). The full p value for comparison of cases and controls in panel b was 0.0009967.

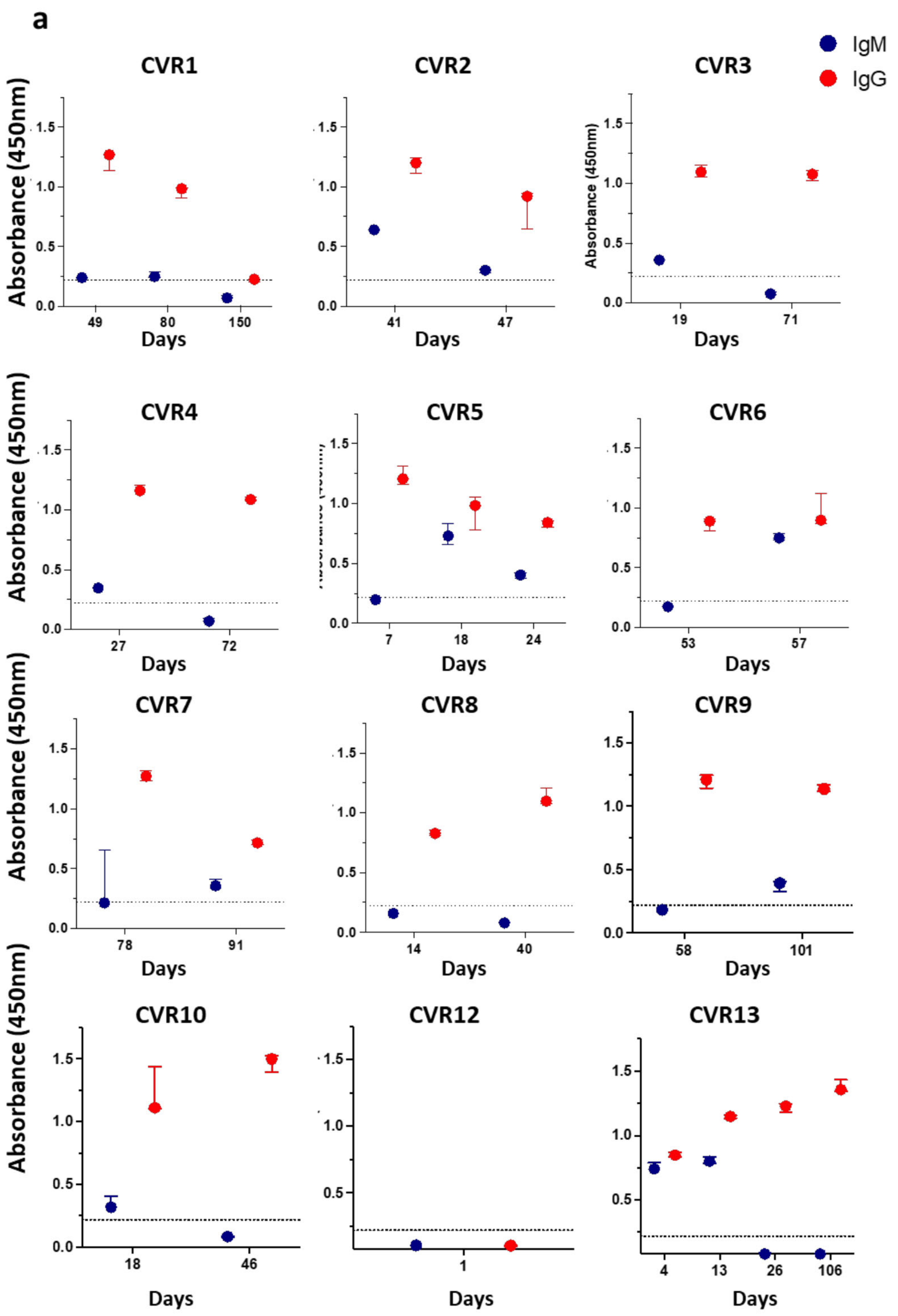
a
IgM
IgG
CVR1
CVR2
CVR3
CVR4
CVR5
CVR6
CVR7
CVR8
CVR9
CVR10
CVR12
CVR13
Absorbance (450nm)
Days

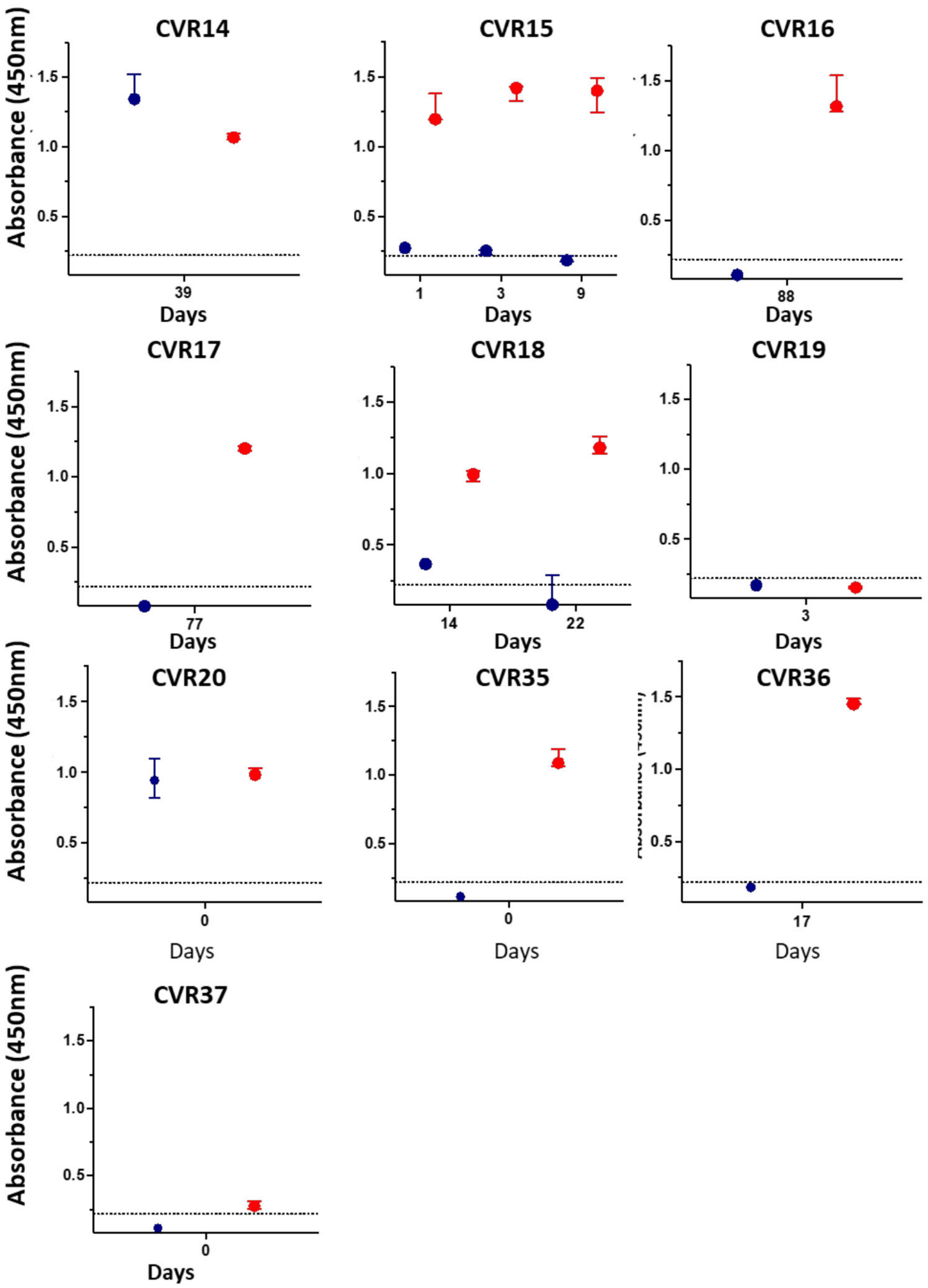
CVR14
CVR15
CVR16
CVR17
CVR18
CVR19
CVR20
CVR35
CVR36
CVR37
Absorbance (450nm)
Days
39
1
3
9
88
77
14
22
3
0
0
17
0
1.5
1.0
0.5

b

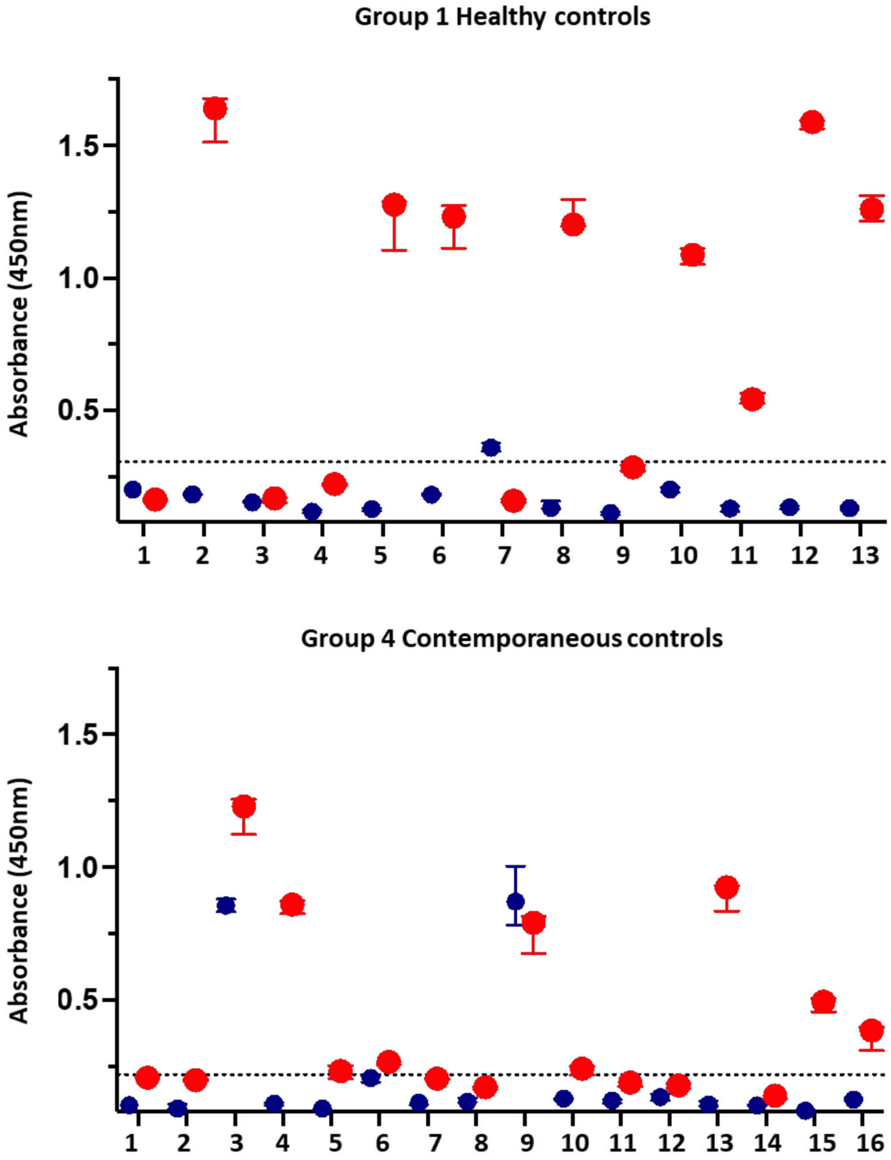

Supplementary Figure 5. AAV2 IgM/IgG levels over time in a) individual cases and b) controls. AAV2 ELISA was carried out using AAV2 virus particles (cat. No. 59462-AAV2, Addgene, UK) at a concentration of $1x10^8$ particles per well in 22 patients from whom longitudinal plasma or serum samples were available. Healthy (Group 1; n=13) and contemporaneous (Group 4; n=16) hospital controls were also tested. All samples were tested in triplicate. Median values (dots) and interquartile range (error bars) are shown.

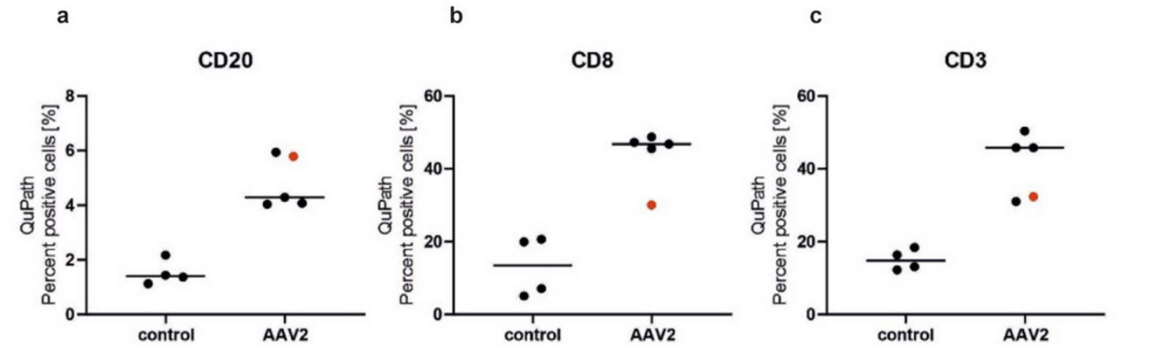

Supplementary Figure 6 | Quantification of immune cells in liver cases and adult controls. The percentage of positively immuno-stained cells were quantified in whole scanned slides of liver tissue. Liver biopsies from cases (n=5) and adult controls (n=4) were analysed (one biopsy from each patient). a) B cells (CD20), **b)** CD8 T cells, and **c)** CD3 T cells were analysed, respectively. The red data point represents data from the explant liver (CVR35). The bar shows the median value.

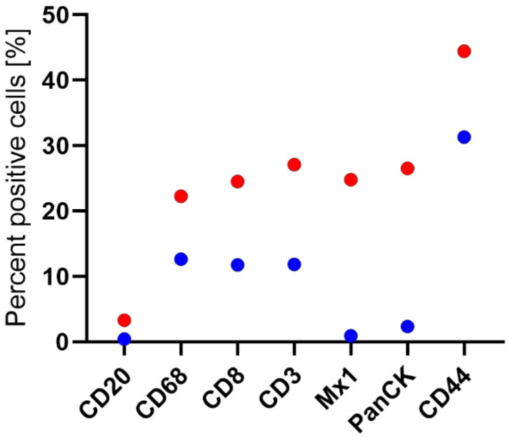

Supplementary Figure 7. Quantification of positively stained cells using CODEX-technology comparing the patient CVR35 (red dots) and a control patient (blue dots) in a single section for each sample after multiplex staining for CD20, CD68, CD8, CD3, Mx1, pan-cytokeratin (PanCK) and CD44.
