# Supplementary material for: Adeno-associated virus 2 infection in children with non-A–E hepatitis
Source: Nature. Author manuscript; Available in PMC 2025 May 12. (PMC7617659; doi:10.1038/s41586-023-05948-2)
Supplement: Supplement Reporting Summary [file EMS203901-supplement-Supplement_Reporting_Summary.pdf]

nature portfolio

Corresponding author(s): Emma Thomson

Last updated by author(s): Dec 14, 2022

Reporting Summary

Nature Portfolio wishes to improve the reproducibility of the work that we publish. This form provides structure for consistency and transparency in reporting. For further information on Nature Portfolio policies, see our Editorial Policies and the Editorial Policy Checklist.

Statistics

For all statistical analyses, confirm that the following items are present in the figure legend, table legend, main text, or Methods section.

n/a	Confirmed	
☐	☒	The exact sample size (*n*) for each experimental group/condition, given as a discrete number and unit of measurement
☐	☒	A statement on whether measurements were taken from distinct samples or whether the same sample was measured repeatedly
☐	☒	The statistical test(s) used AND whether they are one- or two-sided<br>*Only common tests should be described solely by name; describe more complex techniques in the Methods section.*
☒	☐	A description of all covariates tested
☐	☒	A description of any assumptions or corrections, such as tests of normality and adjustment for multiple comparisons
☐	☒	A full description of the statistical parameters including central tendency (e.g. means) or other basic estimates (e.g. regression coefficient) AND variation (e.g. standard deviation) or associated estimates of uncertainty (e.g. confidence intervals)
☐	☒	For null hypothesis testing, the test statistic (e.g. *F*, *t*, *r*) with confidence intervals, effect sizes, degrees of freedom and *P* value noted<br>*Give P values as exact values whenever suitable.*
☒	☐	For Bayesian analysis, information on the choice of priors and Markov chain Monte Carlo settings
☒	☐	For hierarchical and complex designs, identification of the appropriate level for tests and full reporting of outcomes
☒	☐	Estimates of effect sizes (e.g. Cohen's *d*, Pearson's *r*), indicating how they were calculated

Our web collection on statistics for biologists contains articles on many of the points above.

Software and code

Policy information about availability of computer code

Data collection

No commercial code was used for data collection in this study

Data analysis

HLA ANALYSIS
The Bridging ImmunoGenomic Data-Analysis Workflow Gaps (BIGDAWG) R package to derive OR and corrected p values for individual HLA alleles.30 Bonferroni corrected p value significance threshold, adjusted for multiple comparisons (168 HLA alleles), was p < 3.0×10−4.

BIOINFORMATICS ANALYSIS
Reads for each sample were first quality checked, Illumina adapters were trimmed using Trim Galore version 0.6.6 (https://github.com/FelixKrueger/TrimGalore), and reads were then mapped to the human genome using BWA-MEM version 0.7.17 (https://github.com/lh3/bwa). Only reads that did not map to the human genome were used for metagenomic analyses. Non-human reads were then de novo assembled using MetaSPAdes version 3.15.5 (https://github.com/ablab/spades) to generate contigs for each sample. Contigs were then compared against a protein database of all NCBI RefSeq organisms (including virus, bacteria, eukaryotes) with BLASTX using DIAMOND version 2.0.15 (https://github.com/bbuchfink/diamond). In addition, non-human reads for each sample were aligned to a small panel of HAdV NCBI RefSeq genomes (HAdV-A, B1, B2, C, D, E, F, 1, 2, 5, 7, 35, 54 as well as HAdV-F41).

STATISTICAL ANALYSIS
Differences between cases and control groups were tested using Fisher's Exact Test for categorical variables and Mann-Whitney (two tailed) for continuous variables. Spearman's rank correlation coefficients were calculated for the relationships between the trajectories of viral load and ALT and bilirubin. We used R studio version 1.2.5033, R version 4.1.2 and GraphPad version 9.0.0 for most statistical analyses. For coronavirus serology experiments, comparisons were carried out with one way ANOVA and Tukey's Multiple Comparison test, carried out in

GraphPad version 8.4.3.

For manuscripts utilizing custom algorithms or software that are central to the research but not yet described in published literature, software must be made available to editors and reviewers. We strongly encourage code deposition in a community repository (e.g. GitHub). See the Nature Portfolio guidelines for submitting code & software for further information.

Data

Policy information about availability of data

All manuscripts must include a data availability statement. This statement should provide the following information, where applicable:

- Accession codes, unique identifiers, or web links for publicly available datasets
- A description of any restrictions on data availability
- For clinical datasets or third party data, please ensure that the statement adheres to our policy

Datasets generated in the current study are appended as Source Data, Extended Data Tables and Supplementary Tables. Data, protocols, and all documentation around this analysis may be made available to academic researchers after authorisation from the independent data access and sharing committee. Clinical data and analysis scripts are available on request to the Independent Data Management and Access Committee at https://isaric4c.net/ sample_access. Restrictions apply to the availability of identifiable clinical data. Due to the relatively small number of cases, de-aggregation of data is potentially disclosive, as is the patient-level line list data. Therefore, a formal data sharing agreement is required for data access. The Independent Data and Material Access Committee considers requests as they arrive; most responses are made within 28 days. Use of clinical samples are also restricted under ethical approvals obtained for their use. Genome sequences are available in GenBank with accession numbers for AAV2: OP019741-OP019749 and for HAdV-F41: OP019750.

Human research participants

Policy information about studies involving human research participants and Sex and Gender in Research.

Reporting on sex and gender

For the case control study, recruited patients were female (n=20) and male (n=12).
Group 1 healthy control subjects were restricted to 13 children recruited in the UK between January 2020 and April 2022 and were age-matched but not sex-matched due to availability of samples (10 male, 3 female; age range 3-5 years).
Group 2 subjects were children (8 male, 4 female; age range 1-4 years) with PCR-confirmed HAdV infection and normal transaminases
Group 3, 33 children (18 male, 15 female; age range 2-16 years) with raised transaminases who were HAdV PCR negative.
Group 4 included 16 residual samples from children from the NHS GG&C biorepository aged <18 years. Further information was not available under ethical protocols for the use of residual biorepository samples.
For the HLA analysis a further 3 cases were recruited to the ISARIC CCP-UK cohort and had HLA typing carried out but further clinical samples and additional clinical data were not available.

Population characteristics

The median age of affected patients was 4.1 years (IQR: 2.7 to 5.5 years) (Table 1). Twenty of the 32 (63%) children were female, and all were of white ethnicity. Eighteen (56%) of the children reported a subacute history 2-12 weeks prior to acute hepatitis, characterised by an initial gastroenteritis-like illness followed by intermittent vomiting, abdominal pain and fatigue. Most of the affected children (23/32) had no other medical conditions: one child had previously received a liver transplant; none of the other cases were immunocompromised and none had received COVID-19 vaccination. All routine blood tests for viral hepatitis, including hepatitis A, B, C, E, acute Epstein-Barr virus (EBV), cytomegalovirus (CMV), human herpes virus (HHV) 6/7 and herpes simplex virus (HSV) were negative

Recruitment

To investigate the aetiology of the acute hepatitis cases, we recruited 32 of the earliest affected children who presented to hospital between 14 March and 4 April 2022 into the International Severe Acute Respiratory and Emerging Infections Consortium (ISARIC) WHO Clinical Characterisation Protocol UK (CCP-UK) [ISRCTN 66726260]7. All cases who fulfilled the case definition and were willing to participate were recruited. For the HLA analysis a further 3 cases were recruited to the ISARIC CCP-UK cohort and had HLA typing carried out but further clinical samples and additional clinical data were not available.
Control samples (Groups 1,2 and 3) were obtained from the Diagnosis and Management of Febrile Illness using RNA Personalised Molecular Signature Diagnosis study cohort (DIAMONDS; https://www.diamonds2020.eu). This study recruited children presenting with suspected infection or inflammation. Patients were recruited with the informed written consent of parents or guardians.

Ethics oversight

32 affected children, who presented to hospital between 14 March and 20 August 2022 and who met the PHS case definition into the International Severe Acute Respiratory and Emerging Infections Consortium (ISARIC) WHO Clinical Characterisation Protocol UK (CCP-UK) [ISRCTN 66726260].7 Ethical approval was given by the South Central–Oxford C Research Ethics Committee in England (13/SC/0149), the Scotland A Research Ethics Committee (20/SS/0028), and the WHO Ethics Review Committee (RPC571 and RPC572).
Control samples (Groups 1-3) were obtained from the Diagnosis and Management of Febrile Illness using RNA Personalised Molecular Signature Diagnosis study cohort (DIAMONDS; https://www.diamonds2020.eu). Patients were recruited with the written informed consent of parents or guardians.
Contemporaneous Scottish surplus plasma and liver biopsy control samples (Control Group 4) from the Diagnostic Pathology/ Blood Sciences archive were obtained with NHS GG&C Biorepository approval (application #717; REC 22/WS/0020). These samples were used without consent following HTA legislation on consent exemption.
Genetic (HLA) control data was obtained using the UK Biobank Resource (project 788; 21/NW/0157). Participants in the UK Biobank have been recruited with written informed consent.

Note that full information on the approval of the study protocol must also be provided in the manuscript.

Field-specific reporting

Please select the one below that is the best fit for your research. If you are not sure, read the appropriate sections before making your selection.

☒ Life sciences ☐ Behavioural & social sciences ☐ Ecological, evolutionary & environmental sciences

For a reference copy of the document with all sections, see nature.com/documents/nr-reporting-summary-flat.pdf

Life sciences study design

All studies must disclose on these points even when the disclosure is negative.

Sample size	All available cases were selected. All available healthy control samples that could be age-matched to cases were obtained from the DIAMONDS cohort (we planned for up to a 1-3:1 ratio of controls:cases). We selected all available control subjects in group 2 (HAdV positive with normal LFTs) from the DIAMONDS cohort. These were all children but were not age matched. We also selected all available control subject samples in group 3 (hepatitis of alternative aetiology) from the DIAMONDS cohort. We used all available residual samples from children from the same time period as cases in group 4.
Data exclusions	We excluded any cases that did not meet the PHS definition for non-A-E paediatric hepatitis on the basis of age (over 10 years of age or with an alternative diagnosis or from whom clinical data was not available).<br>We excluded 5 plasma samples in the case control study from the NGS analysis of herpesviruses because during nucleic extraction in the relevant clinical laboratory, murine cytomegalovirus (CMV) had been used as an extraction control. This was not used for other sample extractions in the case control study. Clinical specimens taken from cases (throat, rectal swab, faeces and liver samples) also had murine CMV added to the samples and were also excluded from the NGS herpes read count analysis. However, all samples were tested for human herpesviruses by PCR.
Replication	PCR experiments were carried out in triplicate, other than GAPDH PCR which was carried out in duplicate or triplicate. Results were highly concordant. There were four AAV2 Ct values that were borderline (traversing the limit of detection). These were considered negative as weakly positive results were not reproducible and read counts for all samples were negative by metagenomic and target enrichment NGS. Next generation sequencing experiments were repeated on separate runs using different methods (metagenomic sequencing and then semi-agnostic target enrichment sequencing). Results were also confirmed by PCR for AAV2, HAdV and HHV6.
Randomization	As described above, all available cases were selected. All available healthy control samples that could be age-matched to cases were obtained from the DIAMONDS cohort (we planned for up to a 1-3:1 ratio of controls:cases). We selected all available control subjects in group 2 (HAdV positive with normal LFTs) from the DIAMONDS cohort. These were all children but were not age matched. We also selected all available control subject samples in group 3 (hepatitis of alternative aetiology) from the DIAMONDS cohort.
Blinding	The first sequencing run was of samples from 5 cases carried out urgently at the request of Public Health Scotland and investigators were not blinded to these as ethical permissions were not in place for the use of control samples. Subsequent runs were carried out when control samples were available and included 4 further cases. These were analysed with blinding of case/control status and then samples were compared to look for viruses present in cases and controls.<br>PCR, serology and histology experiments were carried out with blinding in place.

Reporting for specific materials, systems and methods

We require information from authors about some types of materials, experimental systems and methods used in many studies. Here, indicate whether each material, system or method listed is relevant to your study. If you are not sure if a list item applies to your research, read the appropriate section before selecting a response.

Materials & experimental systems

n/a	Involved in the study	
☐	☒	Antibodies
☒	☐	Eukaryotic cell lines
☒	☐	Palaeontology and archaeology
☒	☐	Animals and other organisms
☒	☐	Clinical data
☒	☐	Dual use research of concern

Methods

n/a	Involved in the study	
☒	☐	ChIP-seq
☒	☐	Flow cytometry
☒	☐	MRI-based neuroimaging

Antibodies

Antibodies used	For AAV2 ELISA, bound human antibody was detected with either anti-human IgM or anti-human IgG (Merck, UK cat no. A9794 and A1543, respectively). Antibodies for IHC are listed below.<br><br>AAntigen Dilution Clone Product code, company Antigen retrieval Detection system

MHCII 1:200 none M0746, Dako/Agilent Pressure cooking; citrate pH6 Envision Dako Agilent
C4d complement 1:100 none Quidel A213 Antihuman C4d ER2 (20) Leica BOND polymer DS9800 and BOND DAB enhancer
CD3 1:100 LN10 Leica NCL-L-CD3-565 ER2 (20) Leica BOND polymer DS9800 and BOND DAB enhancer
CD4 1:200 1F6 Leica NCL-L-CD4-368 ER2 (20) Leica BOND polymer DS9800 and BOND DAB enhancer
CD8 1:50 4B11 Leica NCL-CD8-4B11 ER2 (20) Leica BOND polymer DS9800 and BOND DAB enhancer
CD20 1:200 L26 Novocastra NCL-L-CD20-L26 ER1 (20) Leica BOND polymer DS9800 and BOND DAB enhancer

Target Ref # Supplier Reporter/Barcode Fluorophore Concentration
CD20 4450018 Akoya Biosciences Bx007 AF750 1/200
CD44 4450041 Akoya Biosciences Bx005 Atto 550 1/100
CD3 4450030 Akoya Biosciences Bx045 Cy5 1/200
PanCK 4450020 Akoya Biosciences Bx019 AF750 1/200
CD31 4450017 Akoya Biosciences Bx001 AF750 1/100
Mx1 M143 Custom made- University Medical Centre Freiburg Bx022 AF 750 1/50
CD8 4250012 Akoya Biosciences BX026 Atto 550 1/200
CD68 4350019 Akoya Biosciences Bx015 Cy5 1/200
CD107a 4350001 Akoya Biosciences Rx006 Cy5 1/200
CD4 4350018 Akoya Biosciences Bx003 cy5 1/200

Validation

For IHC, C4d was validated on Kidney with acute rejection versus normal kidney tissue. Validation policies and procedures were carried out in accordance with ISO accreditation ISO 15189. CD3, CD4, CD8 and CD20 were validated with normal tonsil control tissue and positive case tissue (lymphoma) from a minimum of 3 cases. Procedures were carried out in accordance with ISO 15189. Test subject tissue for these cases was compared with normal controls and negative controls.
For the CODEX analysis, we carried out validation of antibodies by 1.) replacing the primary antibody with isotype serum and 2.) checking in each section that stained cells had the morphology of the cell to be stained, e.g. MHCII macrophage-like cells close to the sinus using concentrations, as recommended by the manufacturer.
